# Inhibition of PFKFB3 in macrophages ameliorates intestinal inflammation by modulating gut microbiota in DSS-induced colitis

**DOI:** 10.1128/msystems.00632-25

**Published:** 2025-12-11

**Authors:** Jia-Hui Gao, Li-Xiang Li, Wei-Jia Li, Xia Wang, Dong-ping Lyu, Xiao-Ran Xie, Shi-Yang Li, Xiu-Li Zuo, Yan-Qing Li

**Affiliations:** 1 Department of Gastroenterology, Qilu Hospital, Shandong University 91623, Jinan, China; 2 Laboratory of Translational Gastroenterology, Qilu Hospital, Shandong University 91623, Jinan, China; 3 Shandong Provincial Clinical Research Center for Digestive Disease, Qilu Hospital, Shandong University 91623, Jinan, China; 4 Advanced Medical Research Institute, Shandong University 726358, Jinan, China; 5 Robot Engineering Laboratory for Precise Diagnosis and Therapy of Gastrointestinal Tumor, Qilu Hospital of Shandong University 91623, Jinan, China; Zhejiang University School of Medicine, Hangzhou, Zhejiang, China

**Keywords:** PFKFB3, ulcerative colitis, macrophages, gut microbiota, *Faecalibaculum*

## Abstract

Phosphofructo-2-kinase/fructose-2,6-biophosphatase 3 (PFKFB3), a key glycolytic enzyme, has attracted increasing attention for its essential roles in various inflammatory responses and immune-related diseases. But the functional relevance and mechanistic basis of the PFKFB3 on ulcerative colitis (UC) remain unclear. Immunohistochemical staining and publicly available data sets were used to analyze PFKFB3 expression in healthy controls (HCs) and UC patients. The role of PFKFB3 on colitis and gut microbiota was investigated by deficiency of PFKFB3 in macrophages (*PFKFB3*^fl/fl^*Lyz2*-Cre) mice. *In silico* meta- and Spearman’s correlation analysis of published high-throughput transcriptomic data analyzed the correlation between PFKFB3 and microbiome-associated genes. The expression of PFKFB3 was significantly upregulated in the colon of both human UC cohorts and colitis mice. Pharmacological inhibition of PFKFB3 by 3-(3-pyridinyl)-1-(4-pyridinyl)-2-propen-1-one (3PO) diminished the severity of colitis. Single-cell RNA sequencing and flow analysis revealed that the upregulated PFKFB3 was predominantly contributed by colonic macrophages. *PFKFB3*^fl/fl^*Lyz2*-Cre mice alleviated experimental colitis in contrast to littermate control (*PFKFB3*^fl/fl^). Concomitantly, *PFKFB3*^fl/fl^*Lyz2*-Cre mice exhibited a remarkably *Faecalibaculum* genus-enhanced microenvironment, which can be horizontally transmitted to co-housed wild-type mice, leading to an attenuation of DSS-induced colitis. However, when antibiotics were administered to *PFKFB3*^fl/fl^*Lyz2*-Cre mice, the transmission effect was lost. By analyzing the UC patient cohort, Spearman’s correlation provided additional evidence for a significant positive correlation between PFKFB3 and microbiota-associated genes expression. This study demonstrated that PFKFB3 deficiency in macrophages could effectively ameliorate colonic inflammation, providing the first evidence that gut microbiota from PFKFB3-deficient mice may represent a novel therapeutic strategy for UC.

**IMPORTANCE:**

PFKFB3 expression was upregulated in the colon of both ulcerative colitis (UC) patients and colitis mice, and this differential expression was predominantly contributed by colonic lamina propria macrophages. Knockout of PFKFB3 in macrophages significantly alleviated DSS-induced colitis. Knockout of PFKFB3 in macrophage mice exhibited a remarkably *Faecalibaculum* genus-enhanced microenvironment, which can be horizontally transmitted to co-housed wild-type mice, leading to an attenuation of DSS-induced colitis; however, when administered to antibiotics, the transmission effect was lost. By analyzing the UC patient cohort, we demonstrated significant positive correlation between PFKFB3 and microbiota-associated gene expression. Our study first elucidates the relationship of PFKFB3 in macrophages with intestinal inflammation and gut microbiota in UC, which may provide a new strategy for the treatment.

## INTRODUCTION

Inflammatory bowel diseases (IBD), encompassing Crohn’s disease (CD) and ulcerative colitis (UC), are chronic and complex disorders characterized by uncontrolled pathogenic inflammation and tissue damage (1). The global incidence of IBD is on the rise (2). Although the precise pathophysiology remains elusive, there is a recognized association with aberrant immune responses in genetically predisposed individuals (3). Under normal conditions, the gut mucosal immune system tolerates bacterial components while retaining the ability to respond to pathogens. Intestinal macrophages are crucial in maintaining immune homeostasis and regulating inflammation (4). In experimental models of IBD, macrophages respond to chemokine signals by migrating to sites of mucosal inflammation, where they express significant levels of pro-inflammatory cytokines and chemokines, thereby activating the mucosal inflammatory response (5). This indicates that macrophages play a crucial role in the pathogenesis of IBD.

Glucose metabolism is acknowledged as a major regulator of macrophage activation and function. In the colonic mucosa of UC patients, severe hypoxia at inflammatory sites shifts macrophages toward glycolysis to meet high energy demands. This metabolic switch is driven by hypoxia-inducible factor-1α (HIF-1α) and key glycolytic proteins, including glucose transporter 1 (GLUT1), hexokinase II (HK2), pyruvate kinase M2 (PKM2), and 6-phosphofructo-2-kinase/fructose-2,6-bisphosphatase 3 (PFKFB3) (6). A recent study has demonstrated that activation of HIF-1α in colonic myeloid cells can accelerate the progression of colitis. Conversely, knockout of HIF-1α can ameliorate the pathological manifestations of colitis (7, 8). These findings indicate that regulators of glucose metabolism are crucial determinants of macrophage and other immune cell functions. PFKFB3, among the four PFKFB enzyme isoforms, exhibits the highest kinase-to-phosphatase ratio (740:1) and catalyzes the synthesis of fructose-2,6-bisphosphate (F-2,6-P2), a potent allosteric activator of 6-phosphofructo-1-kinase (PFK-1), a key glycolysis rate-limiting enzyme (9). Studies have shown that PFKFB3 can be upregulated by environmental signals such as inflammation, triggering inflammatory cell activation (10–12). Additionally, research indicates that homozygous loss of PFKFB3 impairs macrophage efferocytosis and exacerbates atherosclerosis (13). Another study suggests that suppressing PFKFB3 expression in macrophages could enhance VEGF-C production, potentially mitigating the inflammatory effects of ischemia/reperfusion injury (14). Given the diverse effects of PFKFB3 across different organs and diseases, future research is warranted to explore its role in macrophages within the context of colonic immune-related diseases.

Increasing evidence implicates the gut microbiota of IBD (15). Animal studies support this relationship, showing that germ-free conditions and broad-spectrum antibiotics cause significant changes in IBD (16, 17). Recent discoveries suggest immune cells play a pivotal role in this response. Host-derived inflammatory responses are key drivers of microbial community composition, and altered responses may contribute to gut dysbiosis (18, 19). Intestinal macrophages, the body’s largest macrophage reservoir, play a crucial role in maintaining intestinal homeostasis by regulating host-microbiota symbiosis (20). Many recent studies have explored the interaction between macrophages and gut microbiota. For example, macrophage depletion increases the relative abundance of the Firmicutes phylum, which have been linked to a reduced risk of colorectal cancer (21). Moreover, macrophages depletion can selectively facilitate the growth of certain bacteria, leading to dysbiosis including increased fecal fungi and enhanced susceptibility to sepsis (22). Despite the significant impact of glycolysis-related molecules on macrophages, their role in macrophage-mediated gut dysbiosis requires further investigation.

In this study, we demonstrated that PFKFB3 ablation in macrophages alleviated experimental colitis by reshaping the microbial community. Mechanistically, PFKFB3 deficiency created a beneficial microenvironment characterized by an increased abundance of the *Faecalibaculum* genus, enabling the remission effect to be transmitted to co-housed wild-type (WT) mice, also exhibiting significant *Faecalibaculum* upregulation. Furthermore, through meta-analysis of differentially expressed genes and correlation analysis, we validated the interaction between PFKFB3 and the microbiota. Our study provides a theoretical basis for developing PFKFB3 in macrophages as a novel therapeutic target for treating IBD.

## MATERIALS AND METHODS

### Analysis of human patient data sets and human cohort

PFKFB3 expression analysis was conducted on colonic mucosal biopsies from UC patients and HC subjects, utilizing data obtained from the Gene Expression Omnibus (GEO) databases GSE73661, GSE38713, and GSE36807. Colonic tissue biopsies were obtained from Qilu Hospital of Shandong University, with informed consent secured from all participants.

### Mice

WT, *PFKFB3*^fl/fl^, and *Lyz2*-cre mice (C57BL/6 background) were obtained from GemPharmatech. Macrophages-specific PFKFB3 conditional knockout (*PFKFB3*^fl/fl^*Lyz2*-Cre) mice were generated by cross-fertilizing the transgenic mice. Both sex- and age-matched littermates were maintained under specific-pathogen-free conditions (12 h light/dark cycle, 26°C) at Shandong University.

### DSS-induced colitis

DSS-induced colitis model was induced using 2.5% dextran sulfate sodium (DSS; MP Biomedicals) in drinking water for 7 days. Mice received an intraperitoneally injection of PFKFB3-specific inhibitor 3-(3-pyridinyl)-1-(4-pyridinyl)-2-propen-1-one (3PO) (DSS + 3PO group, 50 mg/kg, HY-19824, MedChemExpress) or solvent (DSS group) from days 2 to 6. For neutrophil deletion experiments, mice were intravenously injected with the anti-mouse Ly6G antibody (α-Ly6G, Bio X Cell) at a dose of 100 µg per mouse starting 1 day before the DSS challenge, followed by additional injections on days 2 and 5. In the antibiotic intervention experiment, mice received a broad-spectrum antibiotic cocktail (0.5 mg/mL vancomycin, 1 mg/mL neomycin, 1 mg/mL ampicillin, and 1 mg/mL metronidazole) throughout the experiment period. After a 14-day treatment with antibiotics, mice were challenged with 2.5% DSS. For fecal microbiota transplantation (FMT) experiments, fresh fecal specimens from littermate *PFKFB3*^fl/fl^*Lyz2*-Cre donor mice were homogenized in sterile PBS at a concentration of 200 mg/mL. Following debris removal, the resulting suspensions were cryopreserved in a 25% glycerol solution for future FMT use. The *PFKFB3*^fl/fl^ mice were randomly divided into three groups: *PFKFB3*^fl/fl^ group, *PFKFB3*^fl/fl^ + Abx group, *PFKFB3*^fl/fl^ + FMT group. To prevent the colonization resistance, *PFKFB3*^fl/fl^ + Abx and *PFKFB3*^fl/fl^ + FMT group mice were received a broad-spectrum antibiotic cocktail (0.5 mg/mL vancomycin, 1 mg/mL neomycin, 1 mg/mL ampicillin, and 1 mg/mL metronidazole) for 14 days prior to intervention, as previously described (23, 24). Following a 48 h antibiotic clearance period, *PFKFB3*^fl/fl^ + FMT group mice received donor microbiota via oral administration of 200 µL thawed bacterial suspension daily for 3 consecutive days. One week post-FMT, all three groups were subjected to 2.5% DSS treatment for 7 days to induce colitis. During the DSS exposure period, FMT was maintained every other day until terminal sacrifice (25). For cohousing experiments, 4 to 5 week and gender-matched WT and *PFKFB3*^fl/fl^*Lyz2*-Cre or littermate *PFKFB3*^fl/fl^ mice were co-housed at 1:1 ratio for 4 weeks then induced colitis. For the cohousing experiments after depletion of intestinal microbiota, *PFKFB3*^fl/fl^*Lyz2*-Cre and littermate *PFKFB3*^fl/fl^ mice were pre-treated with antibiotics cocktail for 2 weeks (0.5 mg/m vancomycin, 1 mg/mL neomycin, 1 mg/mL ampicillin, and 1 mg/mL metronidazole) (23, 24) and then co-housed with age- and gender-matched WT mice for 4 weeks followed by 7 consecutive days DSS-induced colitis. Body weight was monitored daily during the ulcerative colitis period. Disease activity index (DAI) scores were calculated based on established criteria (26): (i) body weight loss (0: none, 1: 1%–5%, 2: 5%–10%, 3: 10%–20%, 4: >20%); (ii) stool consistency (0: normal, 1 and 2: loose stool, 3 and 4: diarrhea); and (iii) occult/gross bleeding (0: negative, 1: +, 2: ++, 3: +++, 4: ++++), divided by 3. Mice were euthanized at the indicated time points, and colons were harvested for length measurement, immune cell analysis, and histology.

### Histological and immunohistochemical analysis

Colonic mucosal biopsies from UC patients (inflamed regions) and HC subjects (descending/sigmoid colon) were fixed in 4% formalin, paraffin-embedded, and sectioned (4 µm). For immunohistochemical analysis, sections were treated with citrate antigen retrieval solution (Dingguo) (20 min boiling), blocked, and incubated with PFKFB3 antibody (1:50, ab181861, Abcam) at 37°C for 1 h. After PBS washes, samples were incubated with enhancer solution for 20 min followed by enzyme-labeled goat anti-mouse/rabbit IgG polymer for 20 min at 37°C, and then samples were washed and visualized using 3,3′-diaminobenzidine and hematoxylin.

Mouse distal colon tissues were fixed in 4% paraformaldehyde for 24 h, paraffin-sectioned (4 µm), and stained with Hematoxylin and Eosin (H&E; Solarbio) for histopathological assessment. Pathology scoring evaluated inflammation infiltration, crypt damage, ulceration, and edema using established criteria (27). Briefly, pathology was scored based on the following criteria: a, inflammation (no infiltration, 0; occasional cell limited to submucosa, 1; significant presence of inflammatory cells in submucosa, limited to focal areas, 2; infiltrate present in both submucosa and lamina propria, limited to focal areas, 3; large amount of infiltrate in submucosa, lamina propria and surrounding blood vessels, covering large areas of mucosa, 4; transmural inflammation [mucosa to muscularis], 5); b, crypt damage (none, 0; some crypt damage, spaces between crypts, 1; larger spaces between crypts, loss of goblet cells, some shortening of crypts, 2; large areas without crypts, surrounded by normal crypts, 3; no crypts, 4); c, ulceration (none, 0; small, focal ulcers, 1; frequent small ulcers, 2; large areas lacking surface epithelium, 3); d, edema (absent, 0; present, 1). Images were acquired with an Olympus microscope.

### Western blotting

Colon tissues were homogenized in ice-cold RIPA buffer (Solarbio) and lysed for protein concentration measurement via a bicinchoninic acid (BCA) protein assay kit (ABP Biosciences). Lysates were denatured in 5× SDS buffer (Beyotime) at 95°C for 10 min, resolved on 10% SDS-PAGE gels, and transferred to polyvinylidene difluoride membranes (Millipore). After blocking with 5% non-fat milk, membranes were incubated overnight at 4°C with PFKFB3 Monoclonal antibody (1:5,000, ab181861, Abcam), followed by HRP-conjugated anti-rabbit IgG (1:5,000, ZB-2301; ZSGB-BIO) for 1 h at room temperature. Protein bands were visualized by enhanced chemiluminescence (Millipore) and quantified using Quantity One (Bio-Rad).

### Quantitative real-time PCR

Total RNA from colon tissues was extracted using TRIzol (Invitrogen) and reversely transcribed into cDNA using HiScript III RT SuperMix for qPCR (+gDNA wiper) (Vazyme). Quantitative real-time PCR (qRT-PCR) was done by using ChamQ Universal SYBR qPCR Master Mix (Vazyme). All the gene-specific primers were synthesized by Sangon. Primer sequences are listed in Table S1. Relative expression levels were normalized to GAPDH mRNA expression and calculated using the 2^−ΔΔCt^ method.

### Quantification of inflammatory cytokines and lactic acid and pyruvate in the colon

Frozen colon samples were homogenized in RIPA buffer (Solarbio) and centrifuged (12,000 × *g*, 4°C, 15 min). Protein concentrations were determined via BCA assay (ABP Biosciences). Il1β, Il6, Tnfα, and Il10 levels were quantified using mouse ELISA kits (Lianke Biotechnology), with lactic acid and pyruvate measured using commercial assay kits (Nanjing Jiancheng).

### Isolation of colonic epithelial cells and lamina propria lymphocytes immune cells and flow cytometry

Colon tissues were dissected into 0.5 cm segments, flushed with ice-cold PBS, and incubated in Hank’s balanced salt solution (HBSS) containing 10 mM HEPES (Gibco) and 2 mM EDTA (Invitrogen) at 37°C for 20 min. After washing with PBS containing 2 mM EDTA, the supernatants were filtered through 100 µm strainers to obtain single-cell suspensions. The remaining tissues were digested in RPMI-1640 (Gibco) with 5% fetal bovine serum (FBS, Gibco), collagenase VIII (1 mg/mL), and DNase (Sigma-Aldrich) (0.1 mg/mL) for 1.5 h at 37°C. The resulting cell suspensions were filtered through 70 µm strainers and fractionated using a 40%–80% Percoll (Sigma-Aldrich) gradient to isolate lamina propria lymphocytes. For flow cytometry, cells were stained with LIVE/DEAD Violet Viability/Vitality Kit (Invitrogen), blocked with CD16/32 antibody (Invitrogen), and stained with the following antibodies: CD45 (30-F11), CD11b (M1/70), Ly-6C (HK1.4), Ly-6G (1A8), F4/80 (BM8), CD11c (N418), CD4 (GK1.5), Tbet (eBio4B10(4B10), GATA3 (L50-823), RORγt (AFKJS-9), IL-17A (TC11 18H10.1), Foxp3 (MF-14), and Lin comprised CD11c (N418), CD11b (M1/70), CD3ε (145-2C11), CD19 (1D3/CD19), CD45R/B220 (RA3-6B2), TER-119 (TER-119), FcεRIa (MAR-1), CD5 (53-7.3), Ly-6G (1A8), and CD16/32 (93). Samples were analyzed on a Gallios flow cytometer (Beckman Coulter), with data processed using FlowJo 10 software (FlowJo LLC).

### Fecal 16S rRNA microbial analysis

Fecal pellets from *PFKFB3*^fl/fl^*Lyz2*-Cre or littermate *PFKFB3*^fl/fl^ mice and after-cohoused WT mice or genetically modified mice were collected and stored at −80°C for 16S rDNA gene sequencing. Genomic DNA was extracted using the FastPure Stool DNA Isolation Kit (Majorbio), evaluated by agarose gel electrophoresis and a NanoDrop2000 spectrophotometer (Thermo Scientific, United States), and subjected to PCR amplification of the 16S V3–V4 region with primers 338F/806R. Purified PCR products (2% gel extraction, Qubit 4.0) were pooled and sequenced on the Illumina Nextseq2000 (Illumina, San Diego, CA, USA).

Raw FASTQ files were processed with fastp version 0.19.6 and merged using FLASH version 1.2.7 (28, 29). High-quality sequences were clustered into OTUs at 97% similarity with UPARSE (30), and taxonomic annotation was performed with the RDP classifier against the Silva v138 database under a 70% confidence threshold (31). Microbial community composition of each sample was analyzed at multiple taxonomic levels. Community composition and inter-sample similarity were analyzed using principal coordinate analysis (PCoA) (Bray-Curtis) and PERMANOVA (Vegan package). Genus-level microbial changes were visualized through heatmaps.

### GEO data collection and analysis of IBD patient

Whole-transcriptome data from mucosal biopsies of IBD patients were downloaded from the GEO website (www.ncbi.nlm.nih.gov/geo/) using the GEO query package in R programming language. Publicly available data sets were used for the analysis (GSE9452, GSE13367, GSE14580, GSE36807, and GSE38713). Integrative meta-analysis of differentially expressed data was carried out using the R package “NetworkAnalyst” (https://www.networkanalyst.ca/NetworkAnalyst/home.xhtml) (32). Gene Ontology enrichment analysis was performed using the R package “DAVID” (https://davidbioinformatics.nih.gov/home.jsp) (33, 34).

### Quantification and statistical analysis

Statistical analysis was carried out using GraphPad Prism 8.0.2 software. Data were expressed as mean ± SD. Normality was verified by Shapiro-Wilk test. Statistical significance was determined by ANOVA or unpaired two-tailed Student’s *t*-tests. Mann–Whitney *U* tests were performed as necessary. *P* < 0.05 was considered statistically significant.

## RESULTS

### PFKFB3 is upregulated in the colon tissue during colitis

**Fig 1:**
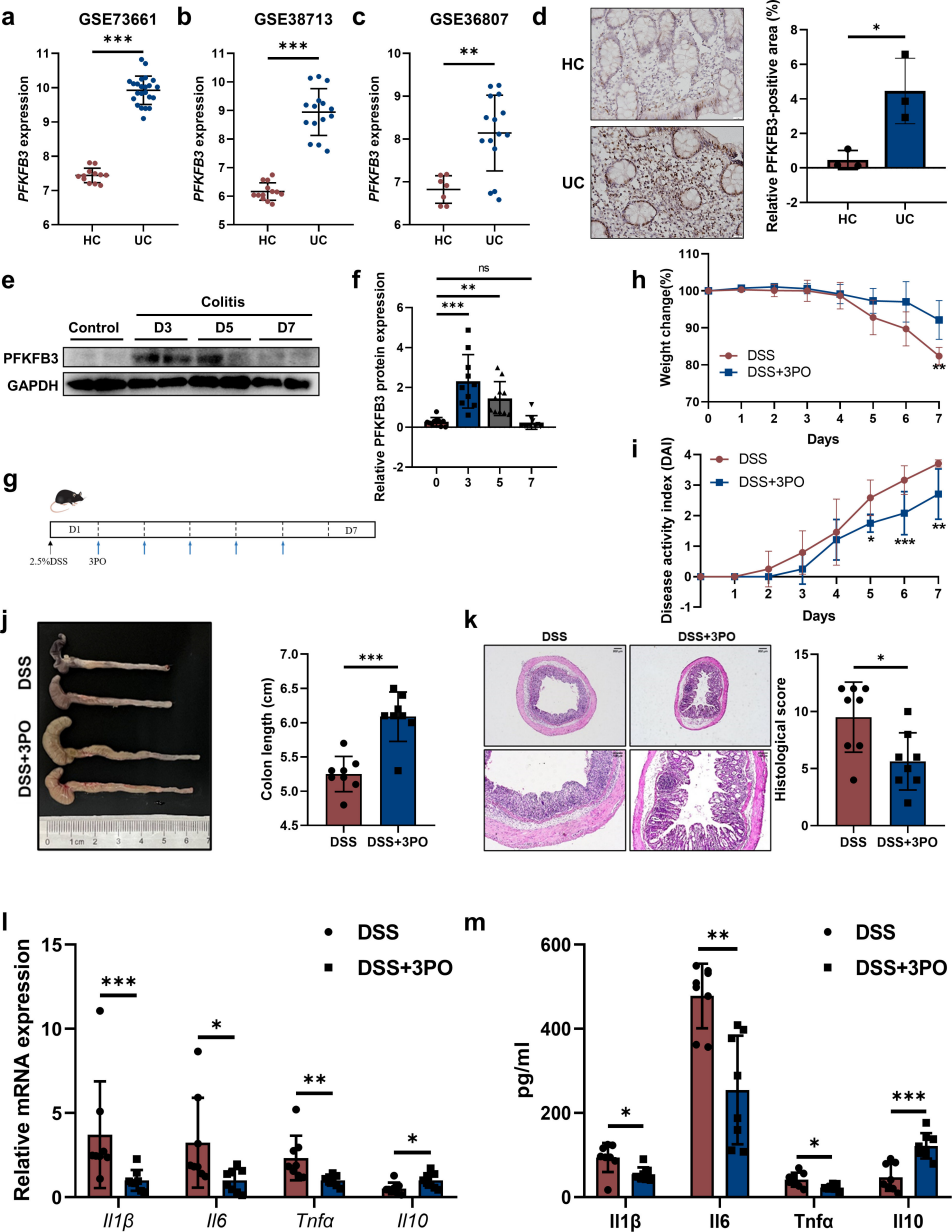
Upregulated expression of PFKFB3 during UC and 3PO alleviates DSS-induced colitis in mice. (**a–c**) PFKFB3 expression was analyzed using GEO data sets, including three independent UC cohort data sets, GSE73661 (**a**), GSE38713 (**b**), and GSE36807 (**c**). HC, Healthy control; UC, UC patients. (**d**) Representative images of PFKFB3 immunohistochemical staining and the quantifications of PFKFB3-positive area measured by ImageJ (*n* = 3 patients). Scale bars, 20 µm. (**e and f**) Representative Western blots and relative expression of PFKFB3 at different time points (*n* = 10). (**g–l**) 3PO intervention experiments (*n* = 8). (**g**) Schematic diagram of the mouse experimental process. (**h**) Body weight. (**i**) DAI scores. (**j**) Colon length comparison. (**k**) Representative images of H&E staining and histological scores. Scale bars, 200 µm or 100 µm for the enlarged figures. qRT-PCR (**l**) and ELISA (**m**) analysis of relative cytokine expression. ns indicates not significant. *P < 0.05, **P < 0.01, and ***P < 0.001.

To evaluate PFKFB3 expression in colitis, we analyzed publicly available data sets from the GEO database. Data revealed significant upregulation of PFKFB3 with active UC across three independent UC cohort microarray analyses (Fig. 1a through c). Consistent with these findings, immunohistochemical analysis confirmed elevated PFKFB3 expression in UC patients compared to HCs (Fig. 1d). Meanwhile, we further verified increased PFKFB3 expression at the protein level in a DSS-induced colitis model, with significant elevations observed on days 3–5, returning to baseline by day 7 (Fig. 1e and f). This transient upregulation may be attributed to severe colonic tissue damage during later stages of DSS-induced colitis, which involves substantial loss of inflammatory and epithelial cells. Collectively, these findings indicate that PFKFB3 expression is markedly enhanced in activated colitis.

### 3PO alleviates DSS-induced colitis in mice

Given that 3PO is a well-established inhibitor of PFKFB3 both *in vivo* and *in vitro* (14, 35), we investigated its role in a DSS-induced murine colitis model. Mice were treated with 2.5% DSS for 7 days, and 3PO (50 mg/kg) was administered intraperitoneally from days 2 to 6 post-colitis induction (Fig. 1g). As shown in Fig. S1, 3PO treatment significantly reduced PFKFB3 protein level during DSS-induced colitis. Moreover, 3PO significantly ameliorated key parameters of colitis severity, including weight loss, DAI scores, and colon shortening (Fig. 1h through j). Histological analysis further revealed reduced epithelial damage and inflammatory infiltration in 3PO-treated mice compared to DSS controls (Fig. 1k). In addition, 3PO-treated mice exhibited lower expression levels of pro-inflammatory cytokines such as Il1β, Il6, and Tnfα, and higher levels of the anti-inflammatory cytokine Il10, as assessed by qRT-PCR (Fig. 1l) and ELISA (Fig. 1m). Levels of lactic acid and pyruvate were also significantly reduced in 3PO-treated mice (Fig. S2). These results demonstrat that systemic administration of 3PO effectively mitigates DSS-induced colitis.

### Expression of PFKFB3 by lamina propria macrophages from colitis mice is increased

**Fig 2:**
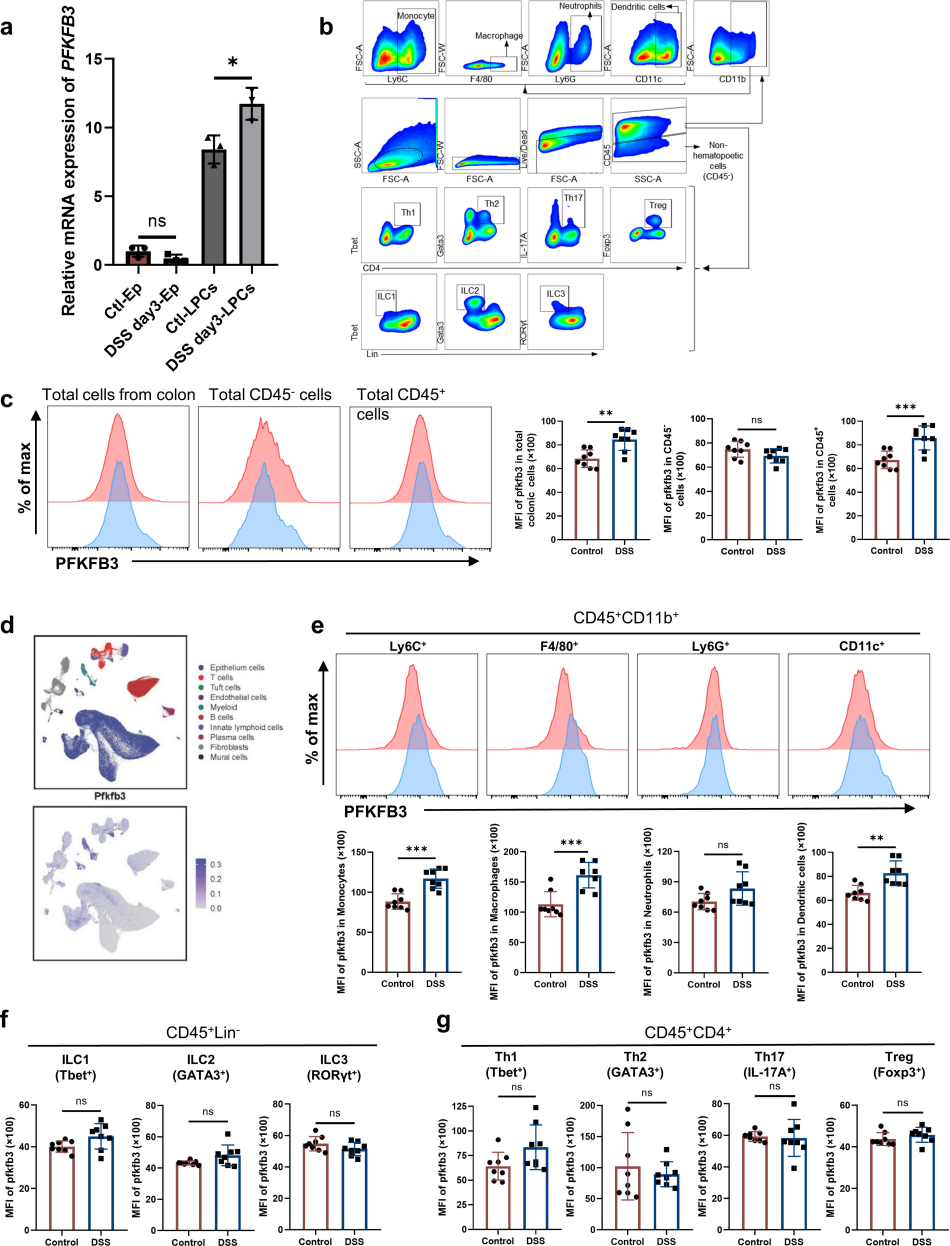
PFKFB3 expression is increased in macrophages of DSS-induced colitis in mice. (**a**) qRT-PCR analysis of PFKFB3 expression in epithelial and lamina propria cells in control and DSS-day 3 mice (*n* = 3). (**b**) The gating strategy. (**c and e–g**) Flow cytometry analysis of PFKFB3 levels in colonic cells at day 0 (Control) and day 3 (DSS) (*n* = 8). Expression of PFKFB3 in CD45^+^ and CD45^−^ cells (**c**); in CD11b^+^Ly6C^+^ (monocytes), CD11b^+^F4/80^+^ (macrophages), CD11b^+^Ly6G^+^ (neutrophils), and CD11b^+^CD11c^+^ (dendritic cells) (**e**); in Lin^-^ ILCs which were further gated into Tbet^+^ (ILC1), GATA3^+^ (ILC2) and RORγt^+^ (ILC3) populations (**f**); in CD4^+^ T cells which were further gated into Tbet^+^ (Th1), GATA3^+^ (Th2), IL-17A^+^ (Th17), and Foxp3^+^ (Treg) T-cell populations (**g**). (**d**) Expression of PFKFB3 in colonic single-cell population of mice. ns indicates not significant. *P < 0.05, **P < 0.01, and ***P < 0.001.

Given the critical role of PFKFB3 in colitis, we prompted further investigation into the cellular localization of PFKFB3 expression within the colon. To this end, qRT-PCR was conducted to identify the source of PFKFB3 overexpression at 3 days post-DSS challenge, a time point with prominent expression differences. Interestingly, our study showed that PFKFB3 expression remained unchanged in epithelial cells (Ep) but was significantly upregulated in lamina propria cells from DSS-treated mice (DSS day3-LPCs) compared to controls (Ctl-LPCs) (Fig. 2a).

Subsequently, flow cytometry was performed to profile PFKFB3-expressing cells (Fig. 2b). Consistent with previous findings, DSS challenge resulted in increased PFKFB3 expression in total colonic cells (Fig. 2c), with marked upregulation specifically in leukocytes (CD45^+^ cells) on the third day of DSS administration, but not in non-hematopoietic cells (CD45^−^ cells) (Fig. 2c), indicating that PFKFB3 induction occurs primarily in leukocytes. Single-cell RNA sequencing further revealed that PFKFB3 was predominantly expressed in myeloid cells, followed by innate lymphoid cells (ILCs), among colonic CD45^+^ leukocytes. Consequently, the study focused on analyzing the expression changes of PFKFB3 in these particular cell types (Fig. 2d). Under inflammatory conditions (DSS day 3), PFKFB3 levels were significantly elevated in monocytes, macrophages, and dendritic cells compared to physiological conditions (DSS day 0), whereas changes in neutrophils were less pronounced (Fig. 2e). In contrast, PFKFB3 expression in ILCs and CD4^+^ T cells did not display significant alterations (Fig. 2f and g).

Among these cell types, macrophages exhibited the highest PFKFB3 expression intensity and greatest upregulation, suggesting a potential colitogenic role of PFKFB3 in macrophages during intestinal inflammation. These findings imply that immunometabolic reprogramming of macrophages under inflammatory conditions may contribute to colitis pathogenesis.

To determine whether the protective effect of 3PO on colitis is independent of the adaptive immune system, we administered 3PO to *Rag2^–/–^* mice, which lack T and B cells. Treated *Rag2*^*–*/^*^–^* mice exhibited less body weight loss and longer colon lengths (Fig. S3a through c). H&E staining further confirmed reduced inflammation in 3PO-treated *Rag2*^*–*/^*^–^* mice (Fig. S3d and e), indicating that 3PO-mediated amelioration of colitis does not require the adaptive immune system.

### PFKFB3 deficiency in macrophages alleviates DSS-induced colitis in mice

**Fig 3:**
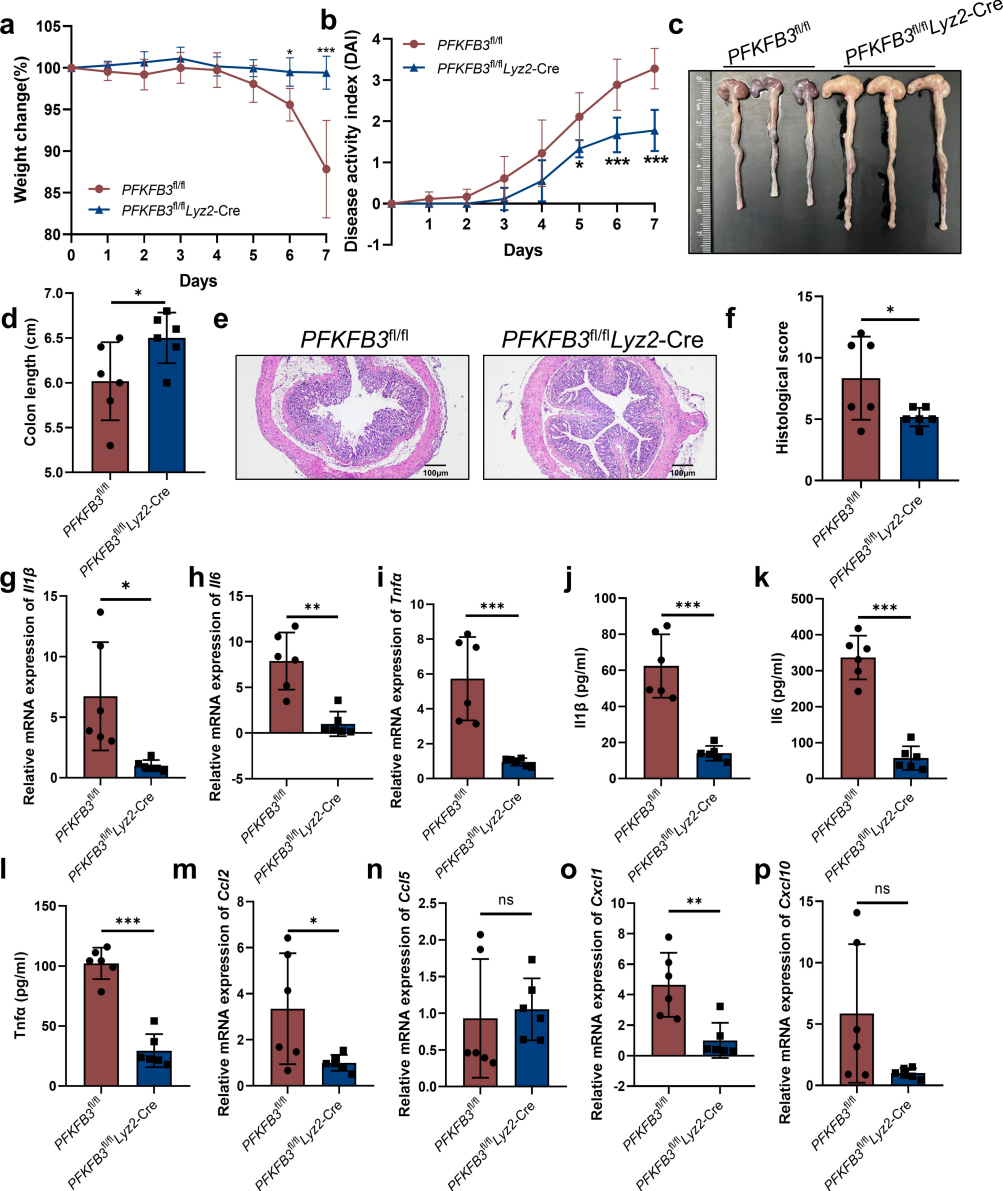
Macrophages-intrinsic PFKFB3 controls DSS-induced colitis. (**a–d**) Weight changes (**a**), DAI scores (**b**), and colon lengths (**c and d**) of *PFKFB3*^fl/fl^ and *PFKFB3*^fl/fl^*Lyz2*-Cre mice. (**e and f**) Representative H&E staining and histology scores. Scale bars, 100 µm. (**g–p**) qRT-PCR and ELISA analysis of relative cytokines expression. *n* = 6. ns indicates not significant. *P < 0.05, **P < 0.01, and ***P < 0.001.

Given the pronounced upregulation of PFKFB3 expression in myeloid cells, particularly macrophages and the central role of innate immune cells in colitis, we generated myeloid-specific PFKFB3 knockout mice by crossing *PFKFB3*^fl/fl^ mice with *Lyz2*-Cre mice (*PFKFB3*^fl/fl^*Lyz2*-Cre) (Fig. S4). Interestingly, compared to littermate controls (*PFKFB3*^fl/fl^), *PFKFB3*^fl/fl^*Lyz2*-Cre mice exhibited reduced weight loss, lower DAI scores, and increased colon length during colitis progression (Fig. 3a through d). Consistently, *PFKFB3*^fl/fl^*Lyz2*-Cre mice also displayed diminished inflammatory cell infiltration and intestinal damage than those of control mice (Fig. 3e and f), along with significantly lower expression of inflammatory cytokines Il1β, Il6, and Tnfα (Fig. 3g through l). Furthermore, qRT-PCR analysis revealed significant decreases in macrophages-associated inflammatory mediators such as ccl2, cxcl1, and cxcl10 (*P* = 0.616), whereas ccl5 expression remained unchanged (Fig. 3m through p).

Since Lyz2-Cre-mediated recombination targets myeloid lineages including macrophages and neutrophils (36, 37), we depleted neutrophils using an anti-Ly6G antibody. Even in neutrophil-depleted mice, PFKFB3 deficiency continued to alleviate colitis phenotype, as indicated by reduced body weight loss, longer colon length, and fewer colon-infiltrating immune cells (Fig. S5a through e), suggesting that the deletion of PFKFB3 in macrophages negatively regulates the colitis phenotypes. These results suggest that PFKFB3 deletion in macrophages, rather than neutrophils, underlies the attenuated colitis phenotype.

### PFKFB3 in macrophages affects the composition of the gut microbiome which plays a crucial role in the susceptibility of DSS-induced colitis

**Fig 4:**
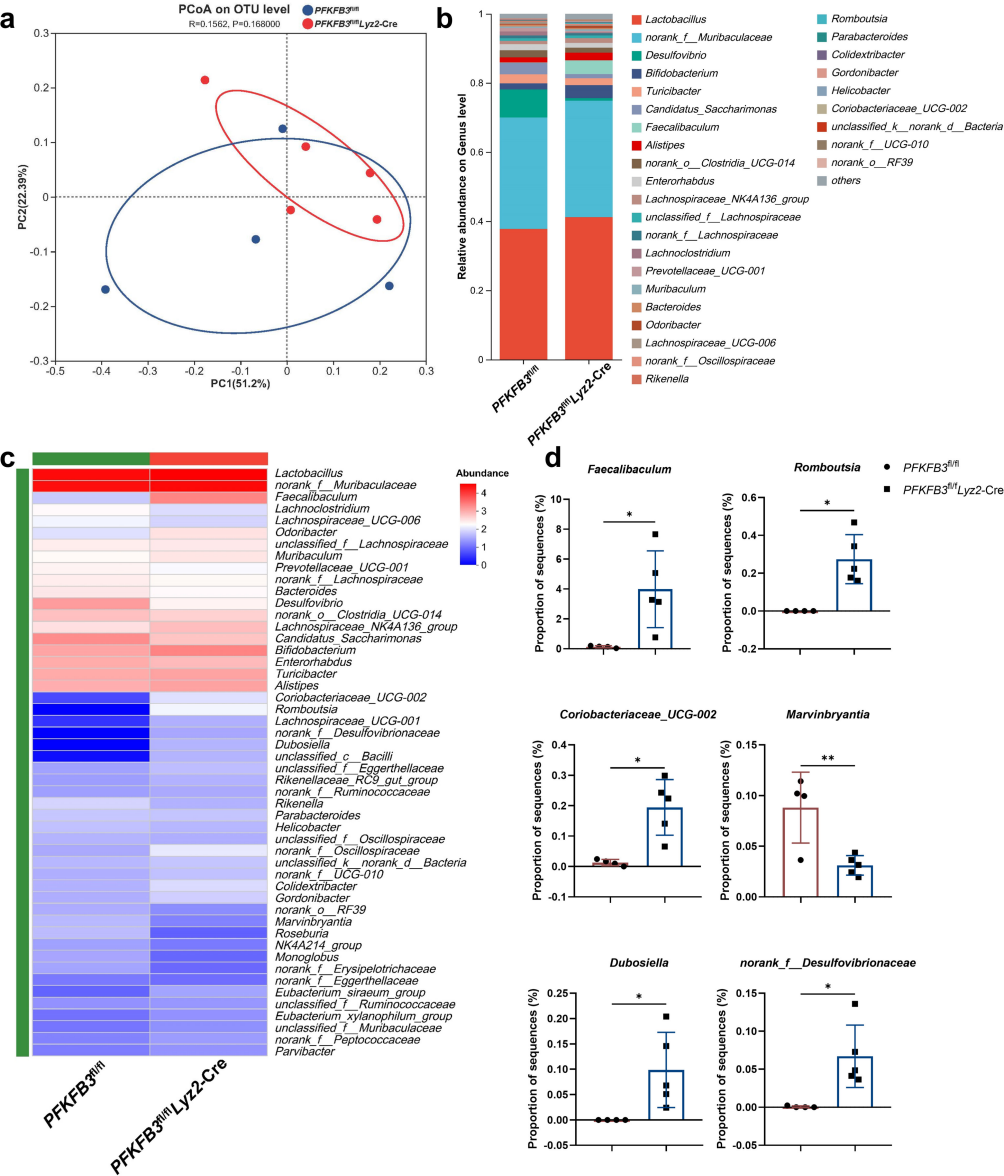
Alterations in gut microbiota in response to PFKFB3 deficiency in macrophages. Feces were collected from *PFKFB3*^fl/fl^*Lyz2*-Cre (*n* = 5) and littermate *PFKFB3*^fl/fl^ (*n* = 4) groups mice to analyze the relative abundance of bacterial communities. (**a**) PCoA analysis. (**b**) Bar plot of microbiota community composition at the genus level. (**c**) Heatmap of taxa in two groups at the genus level. (**d**) Differential abundance of the representative bacteria. *P < 0.05, **P < 0.01.

In the UC colon mucosa, macrophages shift toward glycolysis, a process driven by HIF-1α and key glycolytic enzymes such as GLUT1, HK2, PKM2, and PFKFB3. PKM2 serves as a biomarker for microbial dysregulation in IBD, and HIF-1α is involved in the adaptation to alcohol-induced microbiota changes (38, 39). Given the role of intestinal macrophages in maintaining host–microbe symbiosis, we investigated whether the reduced colitis severity in *PFKFB3*^fl/fl^*Lyz2*-Cre mice was related to alterations in the gut microbiota using 16S rRNA sequencing. Stool samples from *PFKFB3*^fl/fl^*Lyz2*-Cre (5 mice) and *PFKFB3*^fl/fl^ (4 mice) littermate mice were analyzed. PCoA revealed distinct microbial composition in *PFKFB3*^fl/fl^*Lyz2*-Cre mice (Fig. 4a). Community analysis indicated that *Lactobacillus* and *norank_f_Muribaculaceae* were dominant in both groups (Fig. 4b) although notable differences were observed at the genus level (Fig. 4b). A heatmap plot of the top 50 bacterial genera confirmed substantial differences between the *PFKFB3*^fl/fl^ littermates and *PFKFB3*^fl/fl^*Lyz2*-Cre mice (Fig. 4c). A *t*-test on the top 50 most abundant bacterial genera showed that *PFKFB3*^fl/fl^*Lyz2*-Cre mice had increased abundance of *Faecalibaculum*, *Romboutsia*, *Coriobacteriaceae_UCG-002*, *Dubosiella* and *norank_f_Desulfovibrionaceae*, and decreased *Marvinbryantia* (*P* < 0.05) (Fig. 4d).

Having demonstrated that the inhibition of macrophage PFKFB3 expression ameliorates gut dysbiosis, we then examined whether 3PO treatment similarly modulates gut microbiota. PCoA at the genus level indicated that 3PO significantly altered microbial structure (Fig. S6a). The relative abundance of major genera in the DSS and DSS + 3 PO groups was presented in Fig. S6b. Heatmap analysis of the top 50 genera confirmed that the 3PO treatment group exhibited significant differences in microbial genera compared to the DSS group (Fig. S6c). Therefore, we conducted a *t*-test on the top 50 most abundant bacterial genera. As illustrated in Fig. S6d, 3PO treatment significantly increased the relative abundance of 16 genera compared to the DSS model group, while 3 genera were decreased. Notably, *Faecalibaculum*, which was also elevated in *PFKFB3*^fl/fl^*Lyz2*-Cre mice, was enriched in the 3PO intervention group. These data suggest that the gut microbiota from PFKFB3-inhibited mice may have differential effects on colitis.

To assess the functional role of the microbiota, we established a mouse model with antibiotic intervention. Mice received an antibiotic cocktail in their drinking water throughout the experiment. After 2 weeks of antibiotic exposure, the mice were challenged with DSS. The alleviation of weight loss, changes in DAI, and shortening of colon length in *PFKFB3*^fl/fl^*Lyz2*-Cre mice were significantly reduced following antibiotic intervention (Fig. S7a through c). Histopathological analysis revealed comparable levels of mucosal erosion, crypt destruction, and inflammatory cell infiltration in both *PFKFB3*^fl/fl^*Lyz2*-Cre and *PFKFB3*^fl/fl^ mice under antibiotic intervention (Fig. S7d and e). Similarly, there were no statistical differences in Il1β and Tnfα levels post-antibiotic intervention although Il6 showed a significant decrease in the *PFKFB3*^fl/fl^*Lyz2*-Cre group (Fig. S7f). These findings suggest that the protective effect of *PFKFB3*^fl/fl^*Lyz2*-Cre on colitis is impaired by depletion of the gut microbiota. To further assess the involvement of the gut flora in alleviating the effect of *PFKFB3*^fl/fl^*Lyz2*-Cre on colitis, we performed FMT from littermate *PFKFB3*^fl/fl^*Lyz2*-Cre donors to *PFKFB3*^fl/fl^ recipients to investigate the effects of *PFKFB3*^fl/fl^*Lyz2*-Cre mice fecal microbiota of DSS-induced colitis (Fig. S8a). Mice in the *PFKFB3*^fl/fl^+FMT group exhibited higher body weight, lower DAI scores, longer colon, reduced epithelial damage, and lower inflammatory cytokines levels compared to *PFKFB3*^fl/fl^ and *PFKFB3*^fl/fl^+Abx group (Fig. S8b through f). These results indicate that *PFKFB3*^fl/fl^*Lyz2*-Cre modulation of the gut microbiota is essential for preventing colitis.

Overall, these findings suggest that PFKFB3 inhibition remodels the gut microbiota toward a less colitogenic composition.

### The amelioration of colitis in PFKFB3^fl/fl^Lyz2-Cre mice can be transmitted to co-housed WT mice and modulate their bacterial community composition

**Fig 5:**
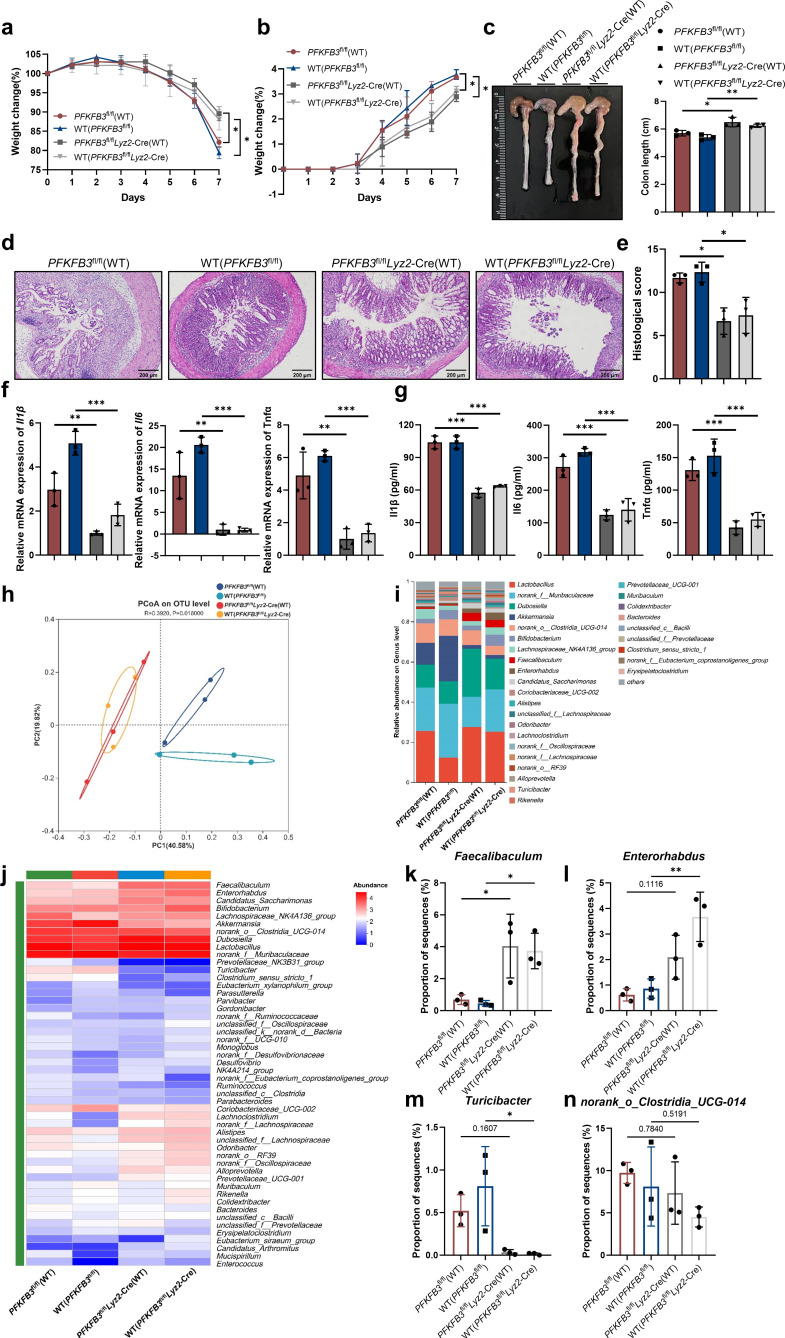
Remission of colitis caused by PFKFB3 deletion is transferable to co-housed WT mice and alterations in bacterial community composition was observed. The body weight (**a**), DAI scores (**b**), colon length (**c**), H&E staining (**d**), and H&E scores (**e**) of *PFKFB3*^fl/fl^(WT), WT(*PFKFB3*^fl/fl^), *PFKFB3*^fl/fl^*Lyz2*-Cre(WT), and WT(*PFKFB3*^fl/fl^*Lyz2*-Cre) mice. Scale bars, 200 µm. (**f**) Relative mRNA gene expression. (**g**) Relative protein expression. (**h**) PCoA analysis. (**i**) Bar plot of microbiota community composition at the genus level. (**j**) Heatmap of taxa at the genus level. (**k–n**) Differential abundance of the representative bacteria. *n* = 3. *P < 0.05, **P < 0.01.

Given that *PFKFB3*^fl/fl^*Lyz2*-Cre mice exhibit colitis resistance and improved microbial profiles, we hypothesized that their altered microbiome reduces colitis susceptibility. To further evaluate this hypothesis, we co-housed two groups of WT (WT(*PFKFB3*^fl/fl^*Lyz2*-Cre; WT(*PFKFB3*^fl/fl^)) mice with either *PFKFB3*^fl/fl^*Lyz2*-Cre (*PFKFB3*^fl/fl^*Lyz2*-Cre(WT)) or *PFKFB3*^fl/fl^ (*PFKFB3*^fl/fl^ (WT)) mice for 4 weeks bred in our vivarium before inducing colitis. Remarkably, cage-matched WT mice and knockout counterparts exhibited similar levels of colitis, although WT mice displayed slightly more severe colon shortening and higher inflammatory cytokine expression, potentially due to differences in maternal inheritance and initial gut conditions. Compared to *PFKFB3*^fl/fl^(WT) mice, the severity of colitis was decreased in *PFKFB3*^fl/fl^*Lyz2*-Cre(WT) group mice as expected (Fig. 5a through g). As well, WT(*PFKFB3*^fl/fl^*Lyz2*-Cre) mice developed less severe colitis compared to WT(*PFKFB3*^fl/fl^) groups, showing reduced weight loss (Fig. 5a), lower DAI scores (Fig. 5b), longer colon length (Fig. 5c), diminished histopathology and intestinal damage (Fig. 5d and e), and lower inflammatory cytokine levels of Il1β, Il6, and Tnfα (Fig. 5f and g). These results conclusively demonstrate that differences in colitis severity between *PFKFB3*^fl/fl^*Lyz2*-Cre and control mice correlate with variations in their intestinal microbiota. Furthermore, the intestinal microbiota of *PFKFB3*^fl/fl^*Lyz2*-Cre mice, characterized by decreased pathogenicity, can be transferred to co-housed WT mice.

To investigate whether the transferable colitis-ameliorating effect observed in *PFKFB3*^fl/fl^*Lyz2*-Cre mice was associated with intestinal microbiota transfer, we collected fecal samples for 16S rRNA sequencing. After 4 weeks of co-housing, cluster analysis revealed that *PFKFB3*^fl/fl^(WT) and WT(*PFKFB3*^fl/fl^), as well as *PFKFB3*^fl/fl^*Lyz2*-Cre(WT) and WT(*PFKFB3*^fl/fl^*Lyz2*-Cre), exhibited similar fecal bacterial phylogenetic structures, while clear distinctions were observed between these two groups (Fig. 5h). Specifically, the fecal microbiota of WT(*PFKFB3*^fl/fl^) mice closely resembled that of *PFKFB3*^fl/fl^(WT) mice, whereas WT(*PFKFB3*^fl/fl^*Lyz2*-Cre) mice showed a profile similar to *PFKFB3*^fl/fl^*Lyz2*-Cre(WT) mice. *Lactobacillus* and *norank_f_Muribaculaceae* remained dominant across groups, consistent with observations in singly housed *PFKFB3*^fl/fl^ and *PFKFB3*^fl/fl^*Lyz2*-Cre mice (Fig. 5i). *Dubosiella* was also a relatively abundant constituent in the fecal microbiota among all groups (Fig. 5i). A heatmap illustrated variations in genera across all groups (Fig. 5j). Notably, consistent with prior findings, co-housing with *PFKFB3*^fl/fl^*Lyz2*-Cre mice for 4 weeks resulted in a significant increase in the relative abundance of *Faecalibaculum* in WT mice, reaching levels comparable to those in *PFKFB3*^fl/fl^*Lyz2*-Cre(WT) mice and exceeding those in WT co-housed with *PFKFB3*^fl/fl^ mice (Fig. 5k). These results suggest that PFKFB3 deficiency in macrophages remodels the gut microbiota, and this effect is transferable to co-housed WT mice, thereby ameliorating colitis. Moreover, compared to the *PFKFB3*^fl/fl^(WT) and WT(*PFKFB3*^fl/fl^) groups, a marked expansion of *Enterorhabdus* was observed in *PFKFB3*^fl/fl^*Lyz2*-Cre(WT) and WT mice co-housed with *PFKFB3*^fl/fl^*Lyz2*-Cre mice. In contrast, relative decreases in *Turicibacter* and *norank_o_Clostridia_UCG-014* were detected in *PFKFB3*^fl/fl^*Lyz2*-Cre(WT) and WT(*PFKFB3*^fl/fl^*Lyz2*-Cre) mice. Therefore, macrophage-specific PFKFB3 deletion improves gut microbial composition by promoting the growth of beneficial bacteria such as *Faecalibaculum*, as corroborated by fecal bacterial sequencing of single-housed *PFKFB3*^fl/fl^*Lyz2*-Cre mice, with evidence supporting that the proliferation of *Faecalibaculum* transfers to co-housed WT mice.

### The transferred alleviative effect on colitis of PFKFB3 knockout in macrophages is resolved with the administration of antibiotics

**Fig 6:**
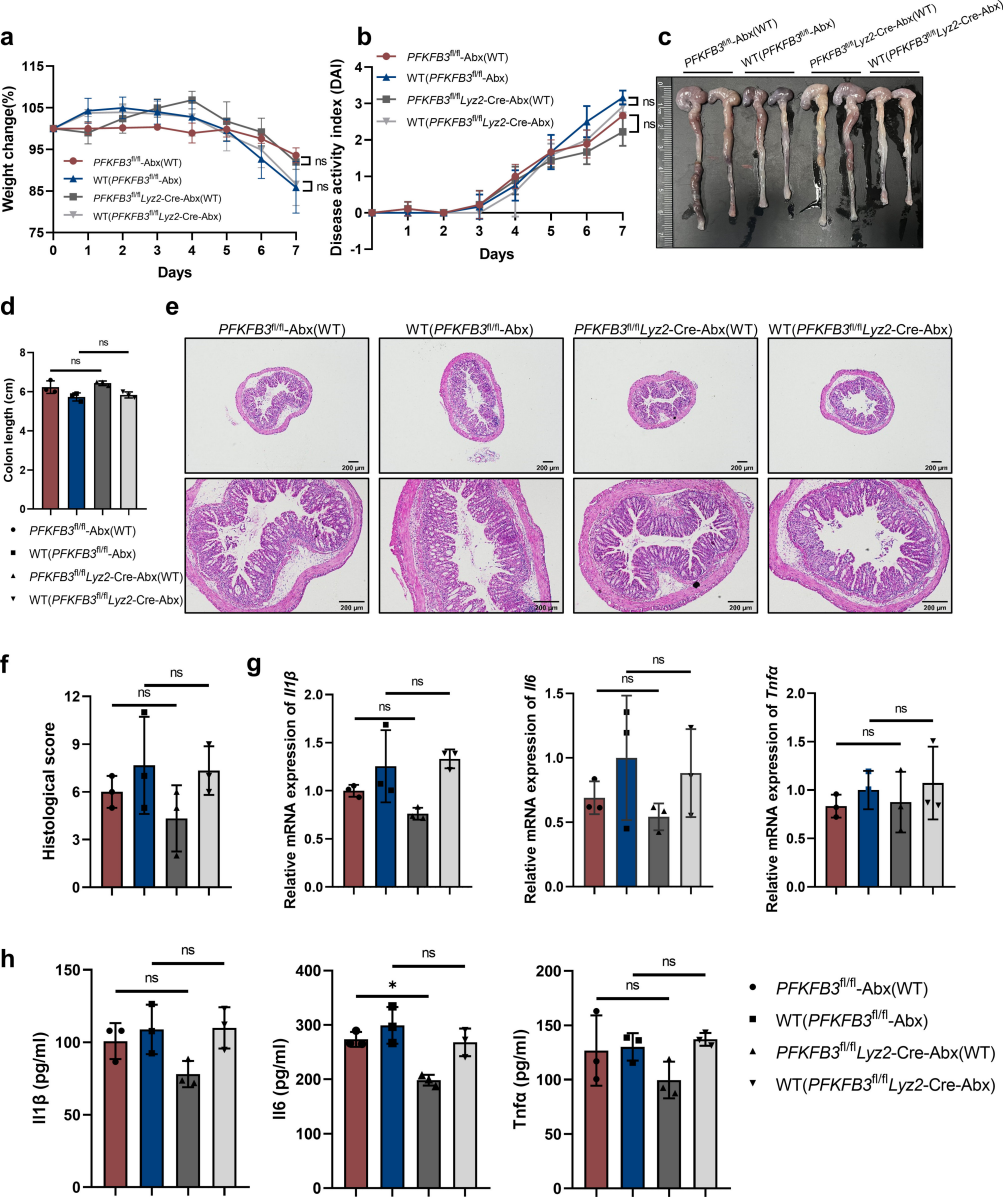
Effects of antibiotic on the ability of *PFKFB3*^fl/fl^*Lyz2*-Cre mice transfering their colitis-alleviating effects to WT cagemates. (**a**) Body weight change. (**b**) DAI scores. (**c and d**) Representative images of the colon and average colon length. (**e**) Representative images of H&E-stained pathological sections. Scale bars, 200 µm. (**f**) Histology score. (**g and h**) Relative expression of cytokines. *n* = 3. ns indicates not significant.

To confirm the role of gut microbes, we investigated the impact of antibiotic treatment on the ability of *PFKFB3*^fl/fl^*Lyz2*-Cre mice to transfer beneficial microbiota to WT mice. For this, *PFKFB3*^fl/fl^*Lyz2*-Cre and littermate *PFKFB3*^fl/fl^ mice were treated with an antibiotic cocktail (0.5 mg/mL vancomycin, 1 mg/mL neomycin, 1 mg/mL ampicillin, and 1 mg/mL metronidazole) for 2 weeks and then co-housed with WT mice for an additional 4 weeks. All groups were subsequently administered 2.5% DSS in their drinking water. Of particular note, antibiotic pretreatment abrogated microbial differences between *PFKFB3*^fl/fl^*Lyz2*-Cre and *PFKFB3*^fl/fl^ mice (Fig. S9a). Of the 41 bacterial genera detected by 16S rRNA analysis, *Faecalibaculum*—a genus previously implicated in potential beneficial effects in our preliminary studies—was not detected. Additionally, *t*-test analysis of these 41 genera demonstrated no statistically significant intergroup differences (Fig. S9b and c). Notably, antibiotic-treated mice exhibited milder symptoms of DSS-induced colitis compared to co-housed WT mice, as evidenced by reduced body weight loss (Fig. 6a), lower DAI scores (Fig. 6b), and milder histological damage (Fig. 6c and d). Strikingly, unlike previous findings, WT mice co-housed with either antibiotic-treated *PFKFB3*^fl/fl^*Lyz2*-Cre or *PFKFB3*^fl/fl^ mice developed similar colitis symptoms, including body weight loss, DAI scores, and colon length reduction (Fig. 6a through d). Histological analysis revealed that WT mice co-housed with different genetically modified antibiotic-treated mice showed comparable DSS-induced inflammatory cell infiltration and crypt destruction (Fig. 6e and f). Furthermore, co-housing did not result in differences in DSS-induced Il1β, Il6, and Tnfα expressions in the colon (Fig. 6g and h). Despite partial protective trends for the histological score and Il1β and Il6 expression, there were no significant differences in the major parameters of colitis severity between the *PFKFB3*^fl/fl^-Abx and *PFKFB3*^fl/fl^*Lyz2*-Cre-Abx groups. Overall, these findings suggest that the gut microbiota in PFKFB3-deficient macrophages mice may involve extensive host-microbial interactions in mitigating DSS-induced colitis.

### Altered gut microbiota-related signaling pathway and genes expression are significantly associated with PFKFB3 expression in IBD patients

**Fig 7:**
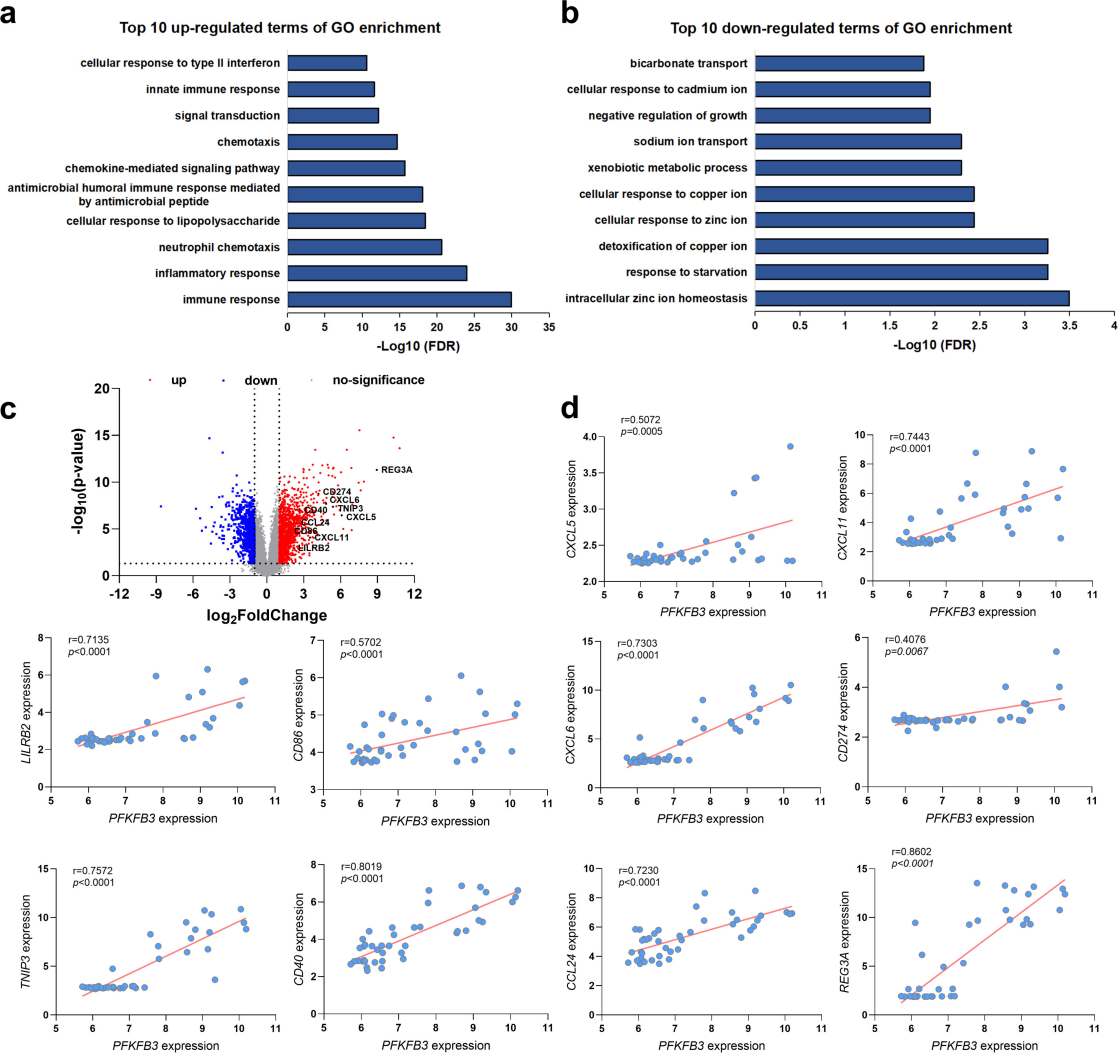
Microbiome-associated signaling is altered in IBD and correlates with PFKFB3 expression. (**a**) Top 10 upregulated terms of GO enrichment of DEGs obtained from in-silico meta-analysis of the previously published high throughput transcriptome from IBD patients. (**b**) Top 10 downregulated terms of GO enrichment of DEGs. (**c**) Hierarchical clustering analysis of DEGs (with a p-adjust threshold of 0.05 and an absolute log 2 [fold change] threshold of 1). (**d**) Representative plots of Spearman correlation analysis of bacteria-associated pathways genes with PFKFB3 in GSE38713.

To evaluate the clinical relevance, we performed an unbiased *in silico* meta-analysis of publicly available high-throughput transcriptomic data from IBD patients. We identified 135 significantly altered canonical pathways, including 125 upregulated and 10 downregulated pathways. Upregulated pathways included processes such as the cellular response to type II interferon, innate immune response, signal transduction, chemotaxis, chemokine-mediated signaling pathway, antimicrobial humoral immune response mediated by antimicrobial peptide, cellular response to lipopolysaccharide, and neutrophil chemotaxis, among others (Fig. 7a). The 10 significantly downregulated pathways included bicarbonate transport, cellular response to cadmium ion, negative regulation of growth, sodium ion transport, xenobiotic metabolic process, and cellular response to copper ion (Fig. 7b). Notably, bacteria-associated diseases and functions were significantly upregulated, such as antimicrobial humoral immune response mediated by antimicrobial peptide and cellular response to lipopolysaccharide. Key genes associated with these signaling pathways included CXCL5, CXCL11, LILRB2, CD86, CXCL6, CD274, TNIP3, CD40, CCL24, and REG3A (Fig. 7c). To further explore these associations, we examined the relationship between PFKFB3 and these genes in IBD patients. Spearman’s correlation analysis using data set GSE38713 revealed a significant positive correlation between PFKFB3 and these genes in UC patients (Fig. 7d). These findings suggest that PFKFB3 upregulation in UC is associated with microbiome-related signaling pathways.

## DISCUSSION

This study exhaustively examines the role of PFKFB3, a pivotal glycolysis regulator, in experimental colitis. Our findings indicate an upregulation of PFKFB3 in colitis-affected colonic tissue, and the deletion of PFKFB3 in macrophages imparts protection against intestinal inflammation through the modulation of gut microbiota.

Glycolysis, consisting of a series of reactions converting glucose to pyruvate, serves as a primary energy source for organisms (40). Current research primarily concentrates on glycolysis’s role in the transition from colitis to cancer, while its impact on the inflammatory stage of colitis remains insufficiently explored (41, 42). PFKFB3, as a critical regulator, has been regarded as a promising target in numerous pathological processes (43–45). In the present study, we demonstrated an enhanced expression of PFKFB3 in colonic tissues of UC patients and DSS-induced colitis. Previous research established connections between PFKFB3 expression and inflammatory processes. In endothelial cells, PFKFB3 knockdown resulted in reduced Tnfα-induced monocyte adhesion, and its inhibition disrupted NF-κB pathway activation (46). Moreover, in macrophages, PFKFB3 influences the activation of the NLRP3 inflammasome and subsequent Il1β release (47). This suggests the essential role of PFKFB3 in the immunomodulatory activities of immune cells. However, its functions in IBD had not been extensively studied. Our study observed that 3PO, a specific PFKFB3 inhibitor, alleviated symptoms and histological damage in DSS-induced colitis. Thus, it is reasonable to infer that 3PO has significant potential to relieve colitis and revealed that PFKFB3 intrinsically aggravated DSS colitis.

Expression levels of PFKFB3 were notably elevated in LPS-activated macrophages, and decreasing PFKFB3 expression constrains macrophage activation and pro-inflammatory responses (47, 48). In the colon, research predominantly targets PFKFB3’s role in colitis-associated colorectal cancer, leaving its function in UC relatively unexplored. Through our study, we affirmed increased PFKFB3 expression in lamina propria cells and macrophages in DSS-induced colitis. Employing conditional knockout mice, we identified a novel functional mechanism whereby PFKFB3 modulates colitis via myeloid cells. However, due to the presence of extensive damage to epithelial cells in the DSS model, we do not rule out the possibility that PFKFB3 in IECs may play some role in inflammatory response and defenses against intestinal pathogen infection. Furthermore, in neutrophil-deficient mice, macrophage-specific PFKFB3 deletion still alleviates colitis, solidifying its role as a positive regulator within macrophages. Thus, our data provide novel insight into the management of PFKFB3 in macrophages during colitis in spite of the potential mechanisms that support the inhibition of pro-inflammatory response remaining partially unknown.

Intestinal macrophages, as the largest pool of macrophages in the body, control the abundance of gut microbiota and limit the absorption of microbial molecules (49, 50). It was demonstrated that the depletion of macrophages increased the relative abundance of the Firmicutes phylum and specifically Lactobacillaceae and Clostridiaceae families (21). In addition, macrophages depletion could selectively facilitate growth of some bacteria to enhanced sepsis (22). Our 16S rRNA sequencing demonstrated that macrophage-specific PFKFB3 deficiency considerably altered intestinal bacterial composition. This led to expansions in the *Faecalibaculum, Romboutsia, Coriobacteriaceae_UCG-002*, *Dubosiella,* and *norank_f_Desulfovibrionaceae* genus, alongside a decrease in the *Marvinbryantia* genus. Mounting evidence underscores *Faecalibaculum*’s critical role in suppressing inflammation by inhibiting *Escherichia-Shigella* and *Oscillibacter* proliferation (51). *Romboutsia,* which was believed to be beneficial for the production of SCFAs, the relative abundance was significantly decreased in the UC groups (52, 53). *Coriobacteriaceae_UCG-002*, as a potential probiotic, was confirmed to be involved in functional intestine recovery in the colitis model (54). Another increased bacteria *Dubosiella* was reported negatively correlated with the pro-inflammatory cytokine gene expression and the relief of colitis (55). However, the effects of *norank_f_Desulfovibrionaceae* remain debated, with studies showing conflicting outcomes in inflammation modulation. One study has shown that *norank_f_Desulfovibrionaceae* is involved in ameliorating the inflammatory response in the ACS model (56), whereas another study has found that *norank_f_Desulfovibrionaceae* may lead to intestinal inflammation (57). Conversely, *Marvinbryantia* proliferation could heighten insulin resistance and Type 2 Diabetes Mellitus (58, 59). The modulation of gut microbiota, accentuated by gut barrier-enhancing bacterial proliferation and diminished inflammation-inducing bacteria, forms a crucial therapeutic mechanism for colitis.

Our co-housing experiments revealed that co-housing *PFKFB3*^fl/fl^*Lyz2*-Cre mice with WT mice for 4 weeks led to relieved colitis in the WT mice. Antibiotic intervention nullified colitis severity differences, underscoring microbiota potential role in colitis remission. Employing 16S rRNA sequencing highlighted substantial distinctions within *Faecalibaculum* genera between *PFKFB3*^fl/fl^*Lyz2*-Cre(WT) and *PFKFB3*^fl/fl^(WT) groups, as well as between WT mice separately co-housed with *PFKFB3*^fl/fl^*Lyz2*-Cre and *PFKFB3*^fl/fl^ groups. These findings suggest that the *Faecalibaculum* genus may be the main beneficial bacteria contributing to the remission colitis and that transfer alleviating effects may be contributed by increased *Faecalibaculum* genus. However, the beneficial impact of increased *Faecalibaculum* necessitates further investigation for direct evidence validation. In addition, both *PFKFB3*^fl/fl^*Lyz2*-Cre(WT) and WT(*PFKFB3*^fl/fl^*Lyz2*-Cre) exhibited enhanced abundances of beneficial *Enterorhabdus* and reduced *Turicibacter* and *norank_o_Clostridia_UCG-014* (60). Although these genera could not be completely matched with the previous sequencing results, WT fecal microbiota might significantly influence fecal microenvironments of knockout mice, inducing dramatic microbial composition alterations.

Significant attention surrounds *Faecalibaculum*’s potential as a next-generation probiotic owing to its promising effects in metabolic disorders. Consistent with our results, numerous studies have reported a negative correlation between *Faecalibaculum rodentium* and IBD. Colonization of an isolated *Faecalibaculum rodentium* has shown it could mitigate colitis in Il10^−/−^ mice (61). Similarly, increased *Faecalibaculum* was significantly negatively correlated with gut permeability (62, 63). Furthermore, its role in downregulating inflammatory proteins through SCFA enrichment is well-documented (64, 65). Our findings demonstrate that macrophage-specific PFKFB3 deficiency correlates with enhanced *Faecalibaculum* genus presence and colitis mitigation. Although we observed a similar effect of WT mice co-housed with *PFKFB3*^fl/fl^*Lyz2*-Cre mice in reversing susceptibility to colitis accompanied by increase of *Faecalibaculum* genus, it should be acknowledged that lack of defined correlation mechanisms limits its clinical applicability. Consequently, further exploration into genetically modified mice interactions with distinct microbiota is warranted.

Next, we examined the potential association between PFKFB3 and these genes in IBD patients. Notably, when we examined the same IBD patient cohort used in the above analysis, Spearman’s correlation demonstrated a significant positive correlation between PFKFB3 and CXCL5, CXCL11, LILRB2, CD86, CXCL6, CD274, TNIP3, CD40, CCL24, REG3A expression. Collectively, the analysis suggests macrophage-specific PFKFB3 loss alters microbiome-related signaling, curbing disease severity.

Furthermore, previous findings noted that increased stromal PFKFB3 exacerbates intestinal inflammation in IBD (66). While our research confirms PFKFB3’s therapeutic potential in colitis, it highlights distinctive mechanistic differences worth examining. Zhou’s findings prioritized fibroblasts involvement during tissue repair, while our work focused on macrophage-mediated inflammatory responses. Several factors contribute to these contrasting conclusions. Cell-specific temporal analysis revealed macrophage-driven PFKFB3 elevation at earlier DSS treatment phases, differing from severe-stage stromal elevation reported by Zhou: Zhou et al. reported that stromal PFKFB3 expression became significantly enhanced only during severe colitis, whereas our data show PFKFB3 upregulation occurs primarily during days 3–5 of DSS treatment. Thus, our study focused on elucidating the cellular sources and functional consequences of PFKFB3 upregulation during colitis’ acute inflammatory phase, while Zhou’s work examined PFKFB3’s role in fibroblast-mediated tissue repair during later stages. Mechanistically, fibroblast inflammatory properties linked to JAK/STAT signaling contrast our identification of protective gut microbiota remodeling in PFKFB3-deficient macrophages. These mechanisms likely represent complementary, cell-type-specific regulatory pathways rather than mutually exclusive processes. The gut microenvironment features extensive crosstalk between stromal and immune cells, suggesting PFKFB3 may coordinate inflammatory responses across cellular compartments through paracrine signaling. Methodological differences further inform these observations. Our macrophage-specific knockout model (*PFKFB3*^fl/fl^*Lyz2*-Cre) and targeted 3PO inhibition enabled precise dissection of immune cell contributions, avoiding the confounding effects of systemic PFKFB3 modulation in whole-animal pharmacological approaches. Collectively, these findings significantly advance our understanding of PFKFB3’s therapeutic potential while emphasizing the importance of context-dependent therapeutic strategies. Our research suggests immune cell-specific inhibition may optimize acute macrophage-dominated inflammation management, while chronic fibrotic stages may warrant stromal-centric adjustments. Furthermore, microbiota transplantation highlights combined metabolic-microbial strategies’ potential in colitis therapy. Investigating PFKFB3 cell-specific contributions throughout various IBD subtypes and progression stages is essential for precision therapeutic advancements.

Macrophage-specific PFKFB3 glycolytic reprogramming emerged as influential in IBD pathogenesis. Our work underscores macrophage-derived PFKFB3’s significant impact on disease progression within DSS-induced colitis models by modifying gut microbiota composition. These findings provide a strong rationale for targeting PFKFB3 as a therapeutic strategy. Within the intestinal inflammatory microenvironment, activated macrophages undergo a pronounced metabolic transition, characterized by the upregulation of PFKFB3 expression. This metabolic reprogramming not only supplies rapid energy to macrophages but also intensifies the inflammatory response through multiple mechanisms. Enhanced glycolysis prompts pro-inflammatory cytokine secretion like Il1β, Il6, and Tnfα. Furthermore, suggested metabolic byproducts sustain inflammation through epigenetic modifications, including histone lactylation (67). This complex metabolic-immune-microbiota interaction advances PFKFB3-focused therapies’ molecular foundations. From a clinical perspective, UC patient colonic tissue displays significant PFKFB3 expression upregulation. Colon cancer studies associate PFKFB3 with lymph node metastasis, TNM staging, and poor prognosis (68). These findings underscore the clinical relevance of this target. Consequently, macrophage-specific PFKFB3 targeting could mitigate acute inflammation and hinder IBD-associated carcinogenesis, presenting multifaceted protective prospects.

Several small-molecule PFKFB3 inhibitors have exhibited significant anti-inflammatory effects in preclinical models, paving the way for IBD therapeutic breakthroughs. For instance, 3PO has demonstrated potent protective effects in models of sepsis-associated acute lung injury. Intraperitoneal administration of 3PO substantially reduced lactic acid levels, inflammatory cell counts, and neutrophil infiltration in bronchoalveolar lavage fluid. It also alleviated pulmonary edema and lowered the expression of key inflammatory cytokines such as Il1β, Il6, Tnfα, and ultimately improved the survival rate of septic mice (69). These findings align with our colitis observations, suggesting promise for inflammation-targeted treatments. More recently, second-generation PFKFB3 inhibitors, including PFK158, exhibit pharmacological vigor in both tumor and inflammation models. In studies of colitis-associated colorectal cancer (CRC), PFK158 was shown to effectively inhibit PFKFB3-driven CRC cell proliferation, migration, and invasion. These effects were closely linked to reduced NF-κB phosphorylation and decreased production of Il1β and Tnfα (70). Given that chronic inflammation drives IBD carcinogenesis, PFK158 benefits high-risk patients by targeting inflammatory and carcinogenic pathways. Importantly, favorable preclinical safety profiles bolster clinical development incentives (71, 72). Despite progress, systemic PFKFB3 inhibition may interfere with normal cellular metabolism, prompting safety challenges. Future efforts should focus on macrophage-specific delivery systems to enhance PFKFB3-targeted therapies’ efficacy and safety.

In summary, our data provide evidence for the role of the PFKFB3 in macrophages in colitis. PFKFB3 macrophage deficiency exerts protective effects by regulating inflammatory responses and gut microbiota. Additionally, PFKFB3 deficiency shifts intestinal bacterial communities, leading to a *Faecalibaculum*-dominated beneficial microenvironment that can be horizontally transmissible to co-housed WT mice, reducing experimental colitis susceptibility. Public database analysis unveiled novel links between PFKFB3 and microbiota-linked IBD patient genes. Our study provides an integrative perspective on intestinal ecological shifts arising from macrophage-specific PFKFB3 deficiency in colitis, revealing its novel regulatory microbiota role. Targeted PFKFB3 therapies offer promising strategies for IBD management.

## SUPPLEMENTAL MATERIAL

Supplemental figures msystems.00632-25-s0001.docx Figures S1 to S9.

Table S1 msystems.00632-25-s0002.xlsx Primer sequences for quantatitive PCR.

ASM does not own the copyrights to Supplemental Material that may be linked to, or accessed through, an article. The authors have granted ASM a non-exclusive, world-wide license to publish the Supplemental Material files. Please contact the corresponding author directly for reuse.
