# Supplementary material for: Inhibition of PFKFB3 in macrophages ameliorates intestinal inflammation by modulating gut microbiota in DSS-induced colitis
Source: mSystems. 2025 Dec 11;11(1):e00632-25. doi: 10.1128/msystems.00632-25 (PMC12817908; doi:10.1128/msystems.00632-25)
Supplement: Supplemental figures — Figures S1 to S9. [file msystems.00632-25-s0001.docx]

**Inhibition of PFKFB3 in macrophages attenuates ulcerative colitis by modulating gut microbiota**

**Jia-Hui Gao ^1,2,3^, Li-Xiang Li ^1,2,3^, Wei-Jia Li ^1,2,3,^, Xia Wang ^1,2,3^, Dong-ping Lyu ^1,2,3^, Xiao-Ran Xie ^1,2,3^, Shi-Yang Li ^1,3,5^, Xiu-Li Zuo ^1,2,3^ and Yan-Qing Li ^1,2,3,4,*^**

1. Department of Gastroenterology, Qilu Hospital, Shandong University, Jinan 250012, China

2. Laboratory of Translational Gastroenterology, Qilu Hospital, Shandong University, Jinan 250012, China

3. Shandong Provincial Clinical Research Center for digestive disease, Qilu Hospital, Shandong University, Jinan 250012, China

4. Robot Engineering Laboratory for Precise Diagnosis and Therapy of Gastrointestinal Tumor, Qilu Hospital of Shandong University, Jinan 250012, China

5. Advanced Medical Research Institute, Shandong University, Jinan 250012, China.

*Correspondence author at: Qilu Hospital of Shandong University, Jinan 250012, China. Yanqing Li: [liyanqing@sdu.edu.cn](mailto:liyanqing@sdu.edu.cn)

**Supplementary Figure Legends**

**Fig. S1**


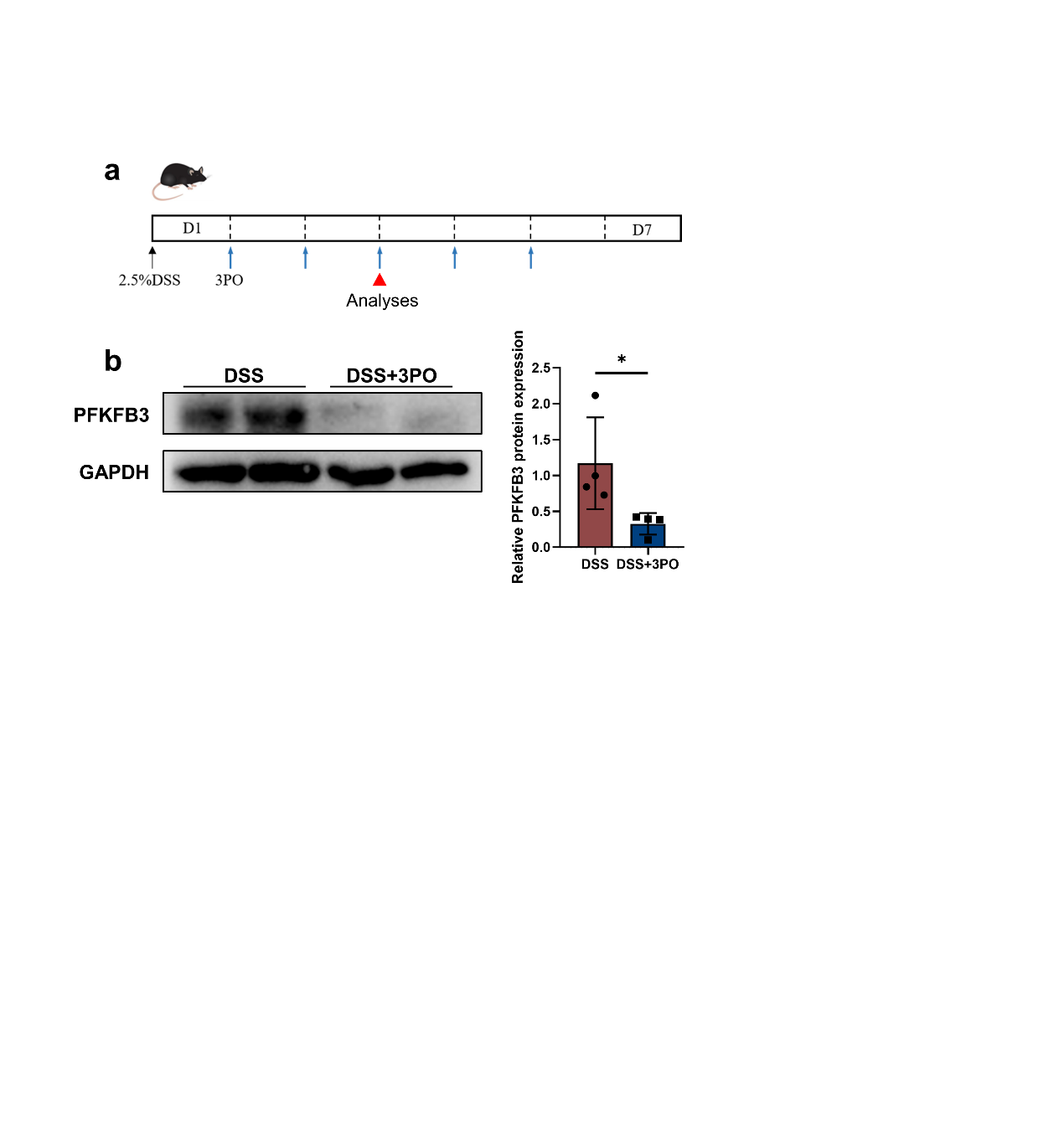


**Fig. S1 3PO decreases PFKFB3 expression in DSS-treated mice colon tissues.** (a) Schematic representation of the experimental design. (b) Western blot analysis and densitometric quantification of PFKFB3 protein levels in DSS or DSS+3PO treatment mice colon. n = 4. **P* < 0.01.

**Fig. S2**


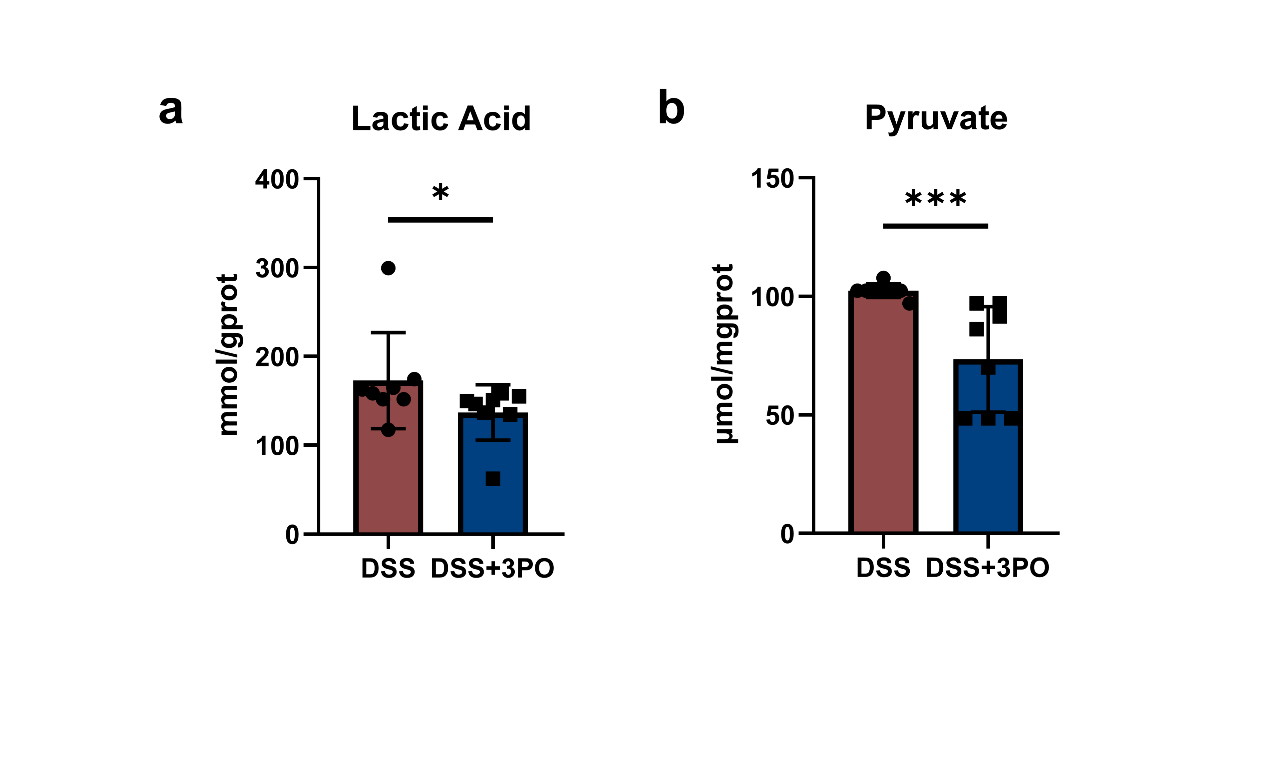


**Fig. S2 3PO inhibits mice colon lactic acid and pyruvate production.** (a and b) Levels of lactic acid and pyruvate production in colon tissues. n=8. **P* < 0.05, ****P* < 0.001.

**Fig. S3**


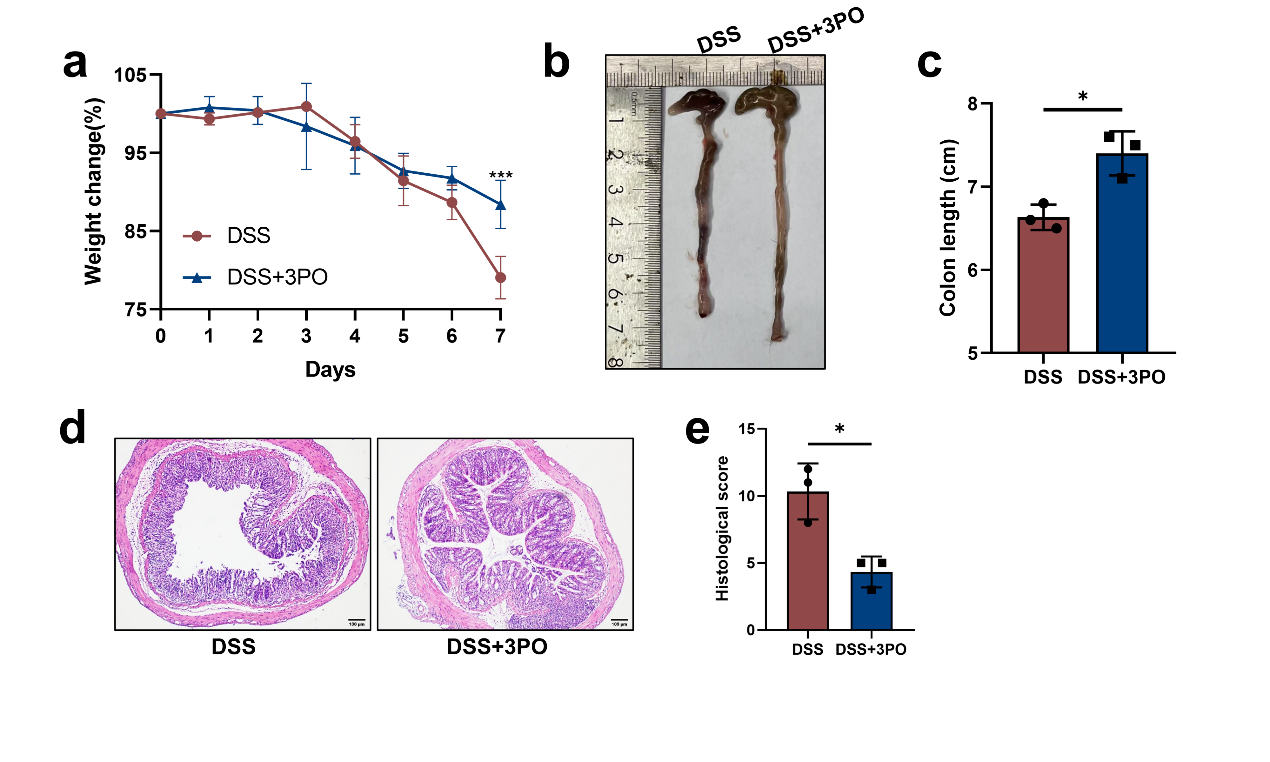


**Fig. S3 Inhibition of PFKFB3 using 3PO alleviates DSS-induced colitis independent of the adaptive immune system.** *Rag2^–/–^* mice were treated with 3PO and 2.5% DSS for analysis 7 days later. (a) Body weight of mice in DSS and DSS+3PO groups. (b and c) Colon length comparison among DSS and DSS+3PO groups. (d and e) Representative images of H&E staining (d) and histological scores of colonic sections (e). Scale bar, 100 μm. n = 3. **P* < 0.05, ****P* < 0.001.

**Fig. S4**

**
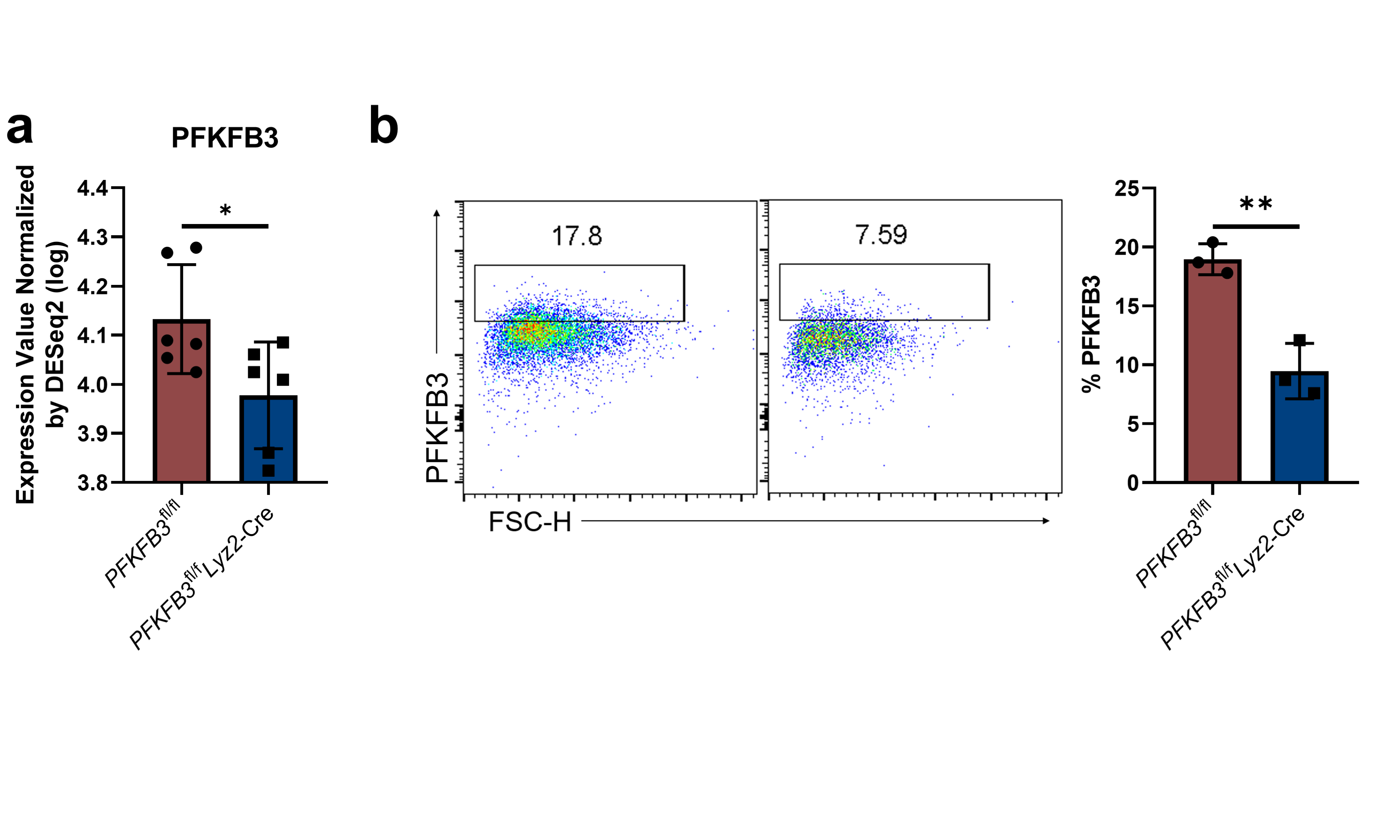
**

**Fig. S4 The expression of PFKFB3 in indicated knockout mice.** (a) The PFKFB3 transcript level in sorted colonic macrophages. n = 6. (b) Flow cytometry analysis of PFKFB3 in colonic CD45^+^CD11b^+^F4/80^+^ macrophages. **P* < 0.05, ***P* < 0.01.

**Fig. S5**


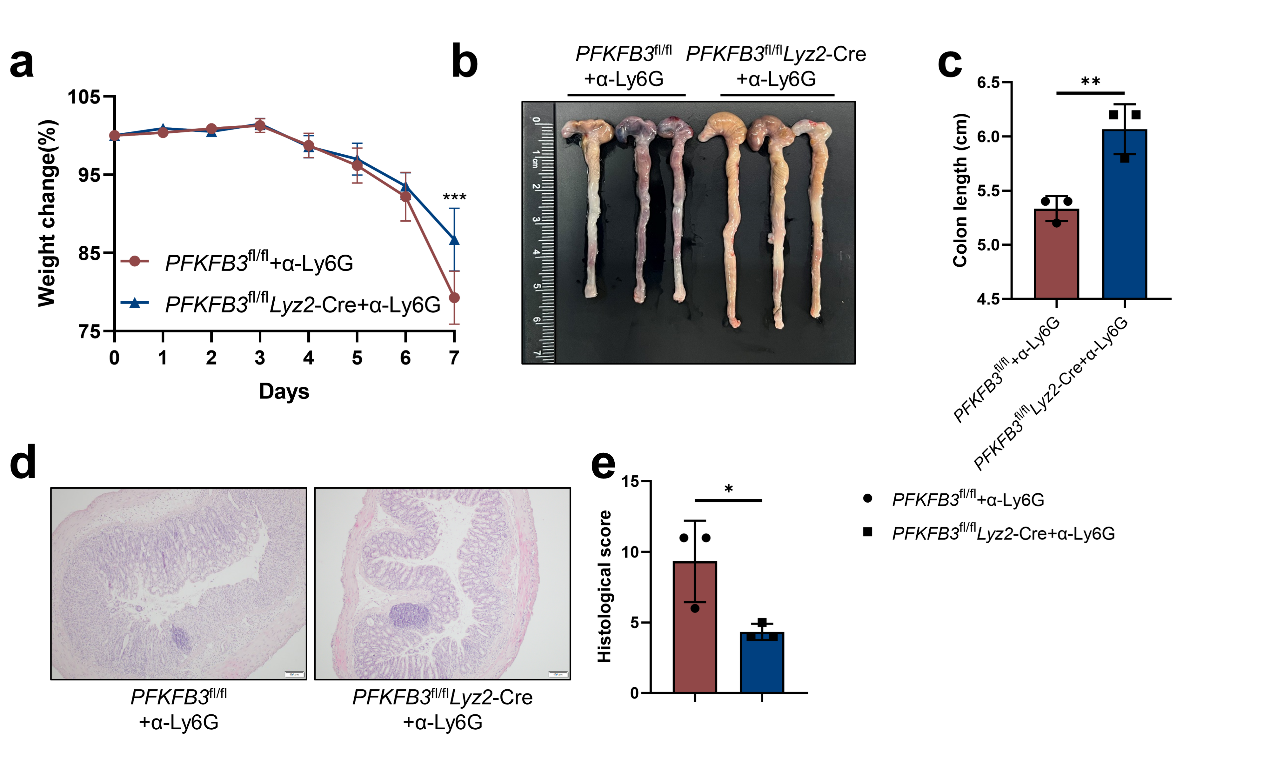


**Fig. S5 PFKFB3 deficiency still alleviates the colitis phenotype in the absence of neutrophils.** *PFKFB3*^fl/fl^*Lyz2*-Cre and littermate control *PFKFB3*^fl/fl^ mice were intravenously injected with anti-Ly6G antibody to delete CD45^+^CD11b^+^Ly6G^+^ neutrophils during DSS treatment. Weight changes (a), colon lengths (b and c) and representative images of H&E staining (d) and histological scores of colonic sections (e) of DSS-treated *PFKFB3*^fl/fl^*Lyz2*-Cre and *PFKFB3*^fl/fl^ mice that were deleted of neutrophils. n=3. **P* < 0.05, ***P* < 0.01, ****P* < 0.001.

**Fig. S6**


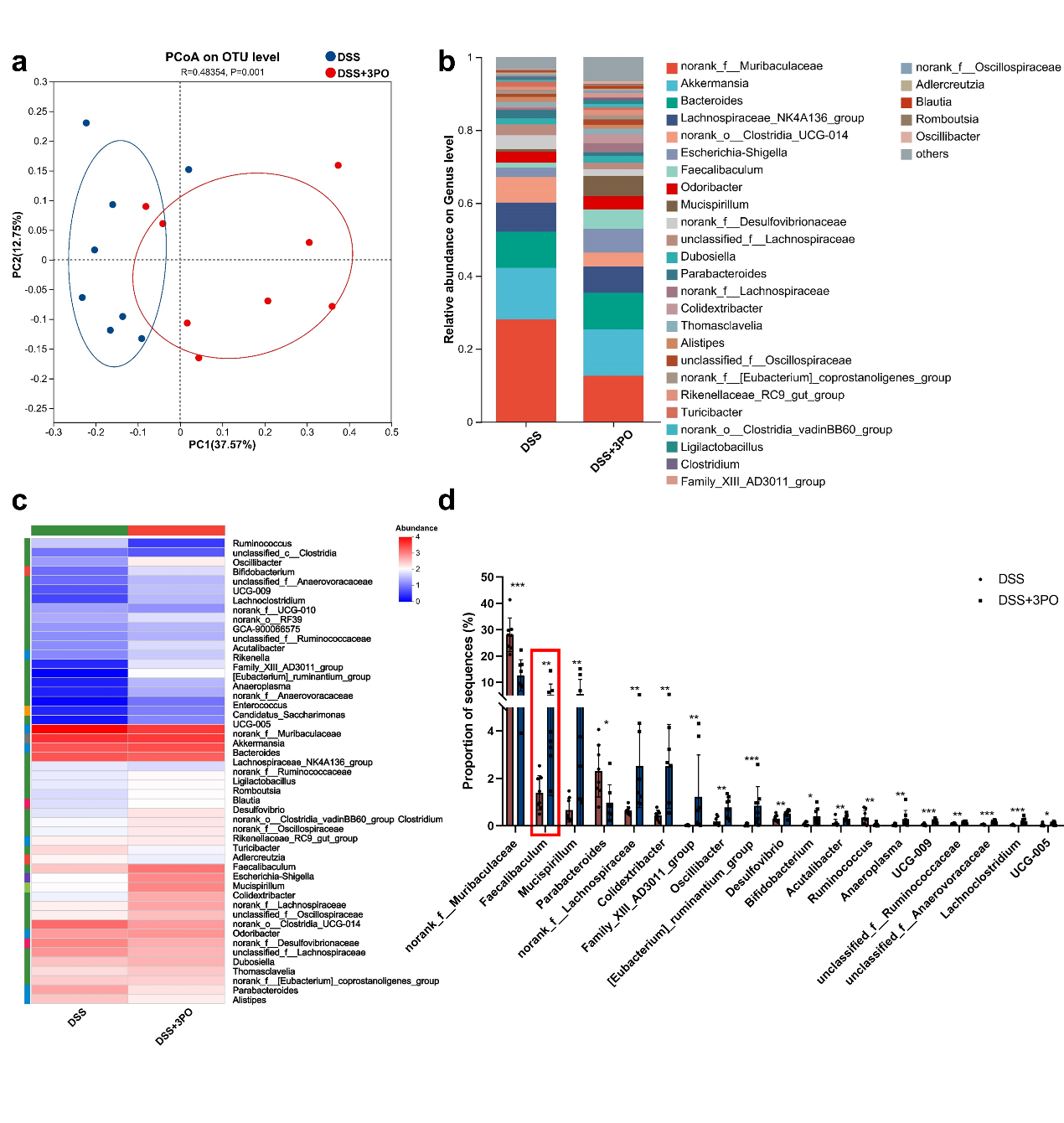


**Fig. S6 Effects of 3PO on the gut microbiota of mice.** (a) Beta diversity of gut microbiota estimated via PCoA analysis on the genera level. (b) Bar plot of microbiota community composition at the genus level. (c) Heatmap of taxa at the genus level. (d) Effects of 3PO on the level of the representative bacteria at the genus level. n=8. **P* < 0.05, ***P* < 0.01, ****P* < 0.001.

**Fig. S7**


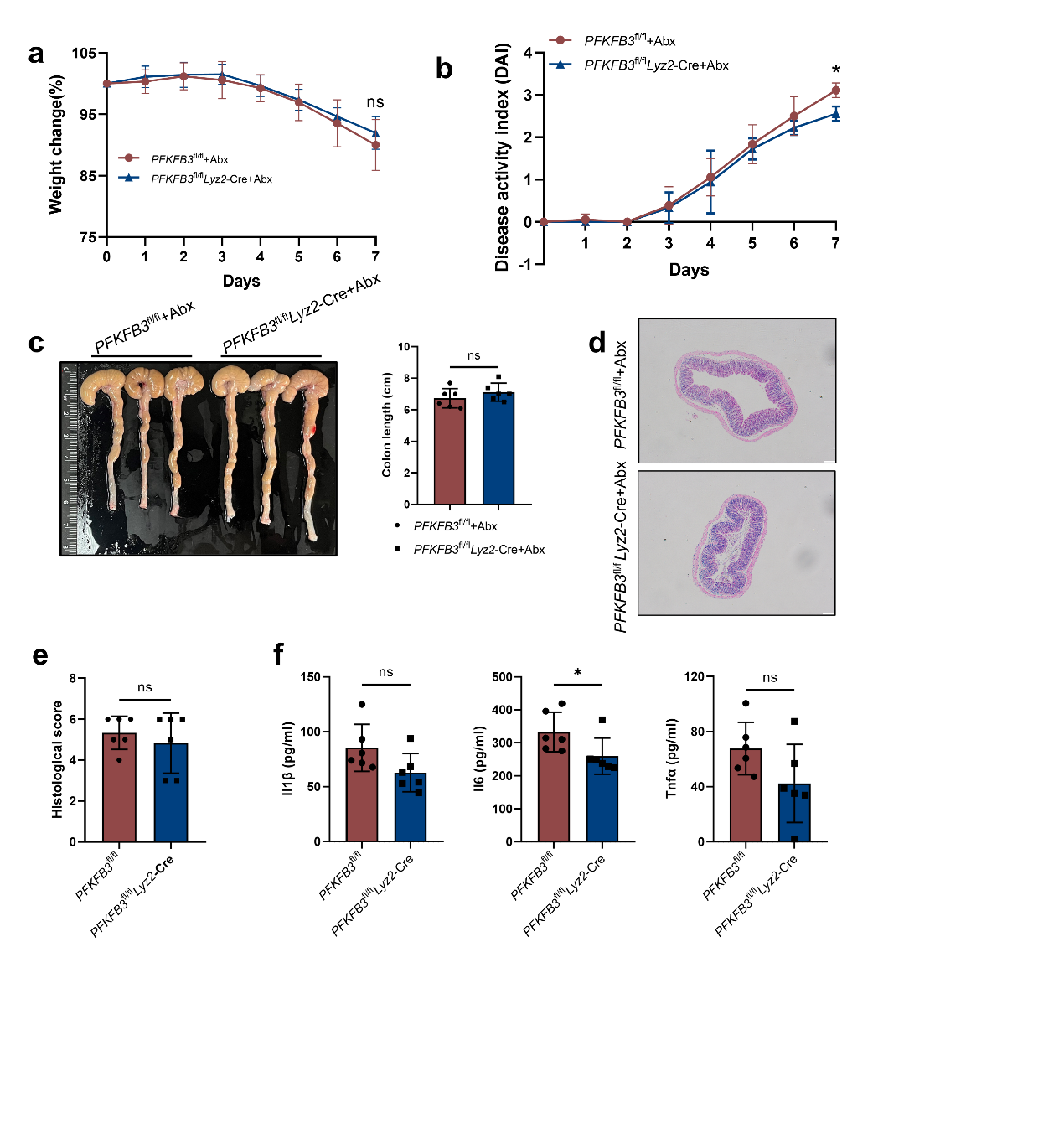


**Fig. S7 Antibiotic intervention alleviates the protective effect of *PFKFB3*^fl/fl^*Lyz2*-Cre mice against colitis.** (a) Changes in body weight. (b) DAI score. (c) Representative colon and colon length. (d) Representative colonic histological images. Scale bar = 200 μm. (e) Colonic histological score. (f) Inflammatory cytokines quantified by ELISA. n=6. **P* < 0.05.

**Fig. S8**


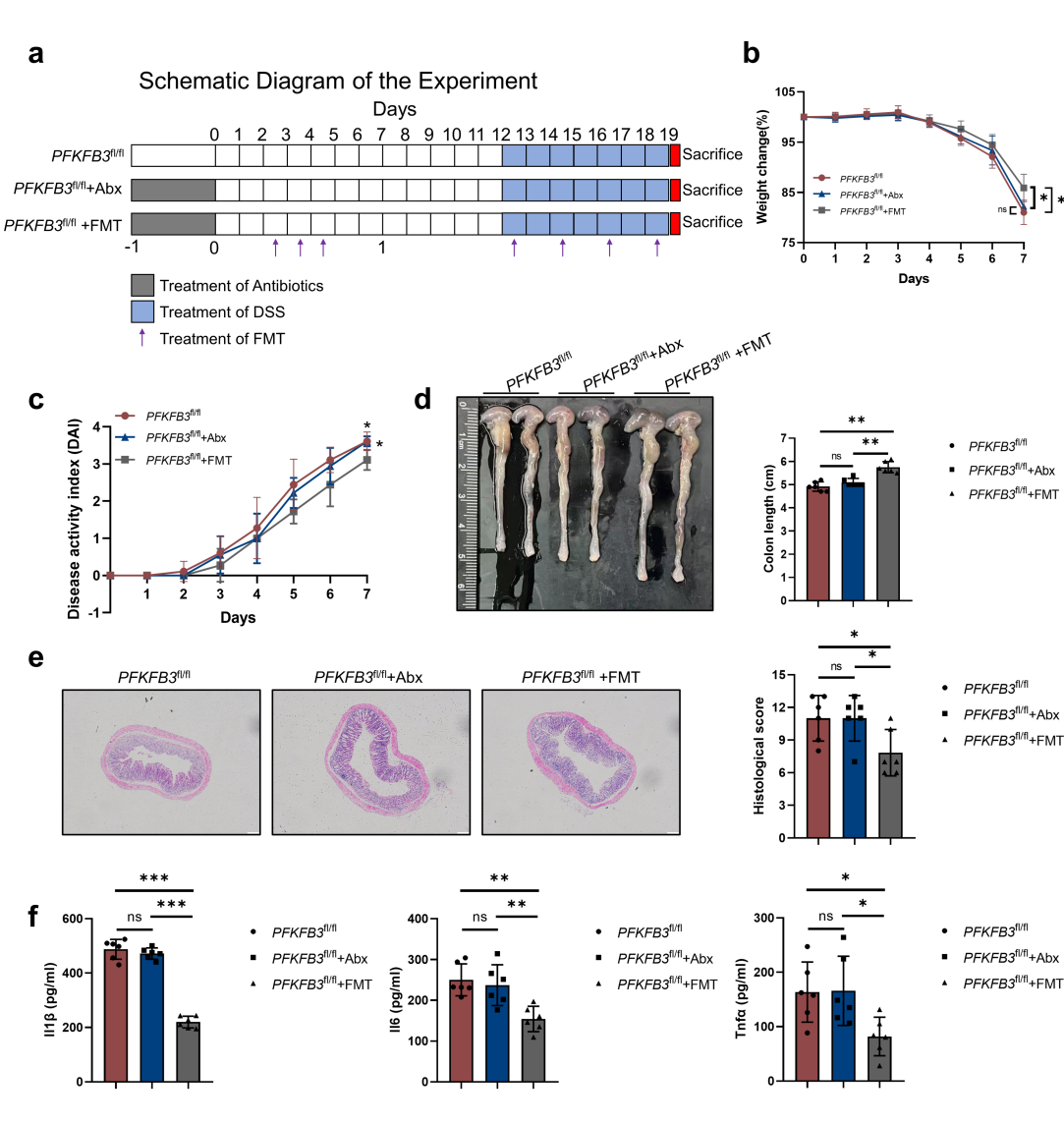


**Fig. S8 FMT from *PFKFB3*^fl/fl^*Lyz2*-Cre mitigates littermate *PFKFB3*^fl/fl^ mice DSS-induced colitis.** (a) Schematic illustration of fecal microbiota transplantation (FMT). (b) Daily body weight change. (c) DAI score of three group mice. (d) Macroscopic observation of the colon and the colon length with statistical analysis. (e) Representative H&E staining images and pathology score. Scale bar = 200 μm. (f) Protein levels of inflammatory cytokines quantified by ELISA. n=6. **P* < 0.05, ***P* < 0.01, ****P* < 0.001.

**Fig. S9**


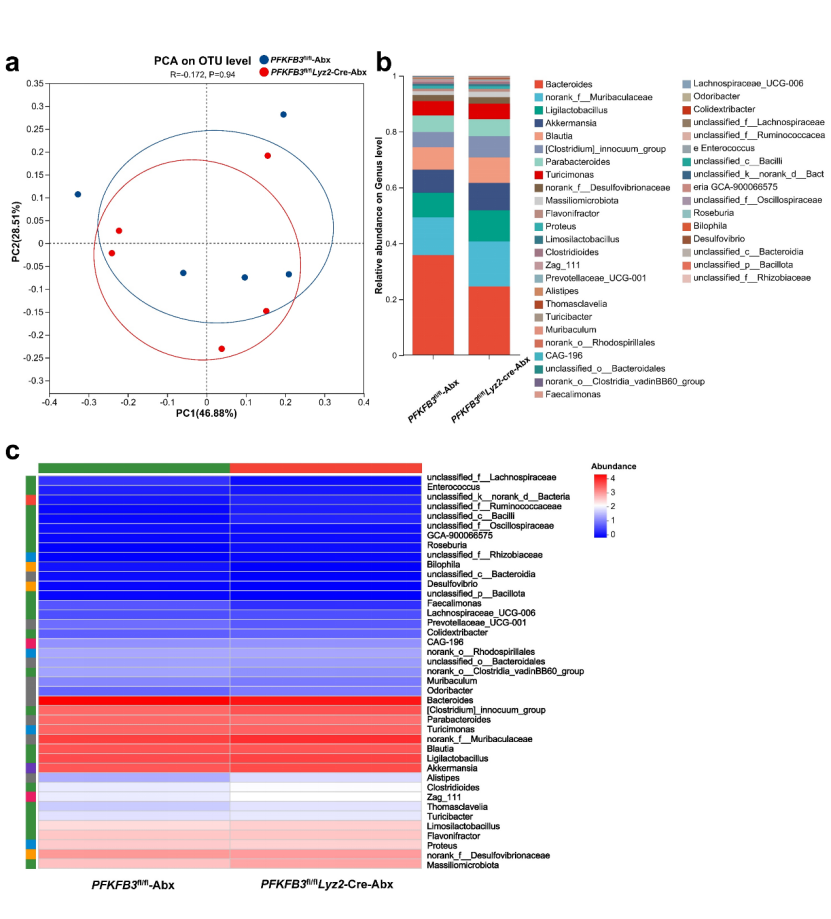


**Fig. S9 Relative abundance of bacterial communities in *PFKFB3*^fl/fl^*Lyz2*-Cre and *PFKFB3*^fl/fl^ treated with antibiotics.** (a) PCoA analysis. (b) Bar plot of microbiota community composition at the genus level. (c) Heatmap of taxa in two groups at the genus level. n=5.
